# Identification of hub genes in papillary thyroid carcinoma: robust rank aggregation and weighted gene co-expression network analysis

**DOI:** 10.1186/s12967-020-02327-7

**Published:** 2020-04-16

**Authors:** Yang Liu, Ting-Yu Chen, Zhi-Yan Yang, Wei Fang, Qian Wu, Chao Zhang

**Affiliations:** 1 Center for Evidence-Based Medicine and Clinical Research, Taihe Hospital, Hubei University of Medicine, No. 32, South Renmin Road, Shiyan, 442000 China; 2 School of Public Health, Xi’an Jiaotong University Health Science Center, Xi’an, 710061 Shaanxi People’s Republic of China

**Keywords:** Papillary thyroid carcinoma, Biomarkers, DNA Methylation, Robust rank aggregation, Weighted gene co-expression network analysis

## Abstract

**Background:**

Papillary thyroid carcinoma (PTC), which is the most common endocrine malignancy, has been steadily increasing worldwide in incidence over the years, while mechanisms underlying the pathogenesis and diagnostic for PTC are incomplete. The purpose of this study is to identify potential biomarkers for diagnosis of PTC, and provide new insights into pathogenesis of PTC.

**Methods:**

Based on weighted gene co-expression network analysis, Robust Rank Aggregation, functional annotation, GSEA and DNA methylation, were employed for investigating potential biomarkers for diagnosis of PTC.

**Results:**

Black and turquoise modules were identified in the gene co-expression network constructed by 1807 DEGs that from 6 eligible gene expression profiles of Gene Expression Omnibus database based on Robust Rank Aggregation and weighted gene co-expression network analysis. Hub genes were significantly down-regulated and the expression levels of the hub genes were different in different stages in hub gene verification. ROC curves indicated all hub genes had good diagnostic value for PTC (except for ABCA6 AUC = 89.5%, the 15 genes with AUC > 90%). Methylation analysis showed that hub gene verification ABCA6, ACACB, RMDN1 and TFPI were identified as differentially methylated genes, and the decreased expression level of these genes may relate to abnormal DNA methylation. Moreover, the expression levels of 8 top hub genes were correlated with tumor purity and tumor-infiltrating immune cells. These findings, including functional annotations and GSEA provide new insights into pathogenesis of PTC.

**Conclusions:**

The hub genes and methylation of hub genes may as potential biomarkers provide new insights for diagnosis of PTC, and all these findings may be the direction to study the mechanisms underlying of PTC in the future.

## Background

Thyroid carcinoma is the most common endocrine cancer, and papillary thyroid cancer (PTC) accounts for the highest proportion of thyroid carcinoma. In recent years, the incidence of PTC has been steadily increasing worldwide, which may be due to a real increase, or may be due to the improvement and widespread use of screening techniques [1]. Increased TSH, autoimmune diseases and inflammation were considered risk factors for thyroid cancer [2]. In recent years, researchers believe that the major carcinogenic event of PTC is the activation of mitogen-activated protein kinase (MAPK) [3]. However, the mechanisms underlying the pathogenesis of PTC have not yet been elucidated. Surgical resection, TSH inhibition therapy and radioactive iodine therapy are the conventional treatment methods for PTC [4]. With these therapeutic approaches, most patients with PTC have a good prognosis, but there were some challenges for both patients and clinicians. The consequence of surgical excision that is one of the important treatments for thyroid cancer may be the increased incidence of hypothyroidism [5]; PTC is often difficult to diagnose because of similarities between malignant and benign nodules, which results in some benign patients having their thyroid removed [6]; treatment of refractory radioiodine differentiated thyroid cancer still faces challenges and some slow-moving tumors were overdiagnosed and overtreated. In terms of molecular therapy, the combined use of immune checkpoint inhibitors and BRAF (especially BRAFV600E) inhibitors is aussichtsreich in the treatment of thyroid cancer and some progress has been made [7]. However, not all tumors have mutations in BRAF [8–10], and other biomarkers are needed. In addition, immunotherapy is associated with immune-related adverse events (autoimmune toxicities) [11]. For example, the use of mAbs anti-cytotoxic T lymphocyte antigen 4 (anti-CTLA-4) and anti-programmed cell death protein-1/programmed cell death ligand-1 (anti-PD-1/PD-L1) causes thyroid dysfunction (including painless thyroiditis and so on) [12] in 10 percent of cancer patients [7]. DNA methylation, which belongs to epigenetics [13], can affect gene expression by affecting the structural stability of chromosomes and the interaction between DNA and proteins [14]. Abnormal levels of DNA methylation had been reported in almost all cancers. The study of DNA methylation is very important for the pathogenesis, early diagnosis and prognosis prediction of tumors and methylation drugs are the darlings of recent targeted therapies for cancer for DNA methylation itself is reversible. The combination of genetic alterations and DNA methylation alterations may improve their clinical value. However, the specific gene map and methylation map of PTC are not complete. Therefore, it is necessary to further understand the underlying biological mechanisms underlying the onset and development of PTC and to identify potential biomarkers for diagnosis. More accurate diagnosis leads to more personalized treatment, which is valuable in further improving patient survival.

As a bioinformatics method that can integrate gene lists of different technology platforms, Robust Rank Aggregation (RRA) is widely used in cancer research [15–17]. This method can reduce noise increase signal while integrating data information of different platforms, which makes the research results more reliable [16]. The weighted gene co-expression network analysis (WGCNA) [18] method is widely used in cancer research, but there is still room for the research of establishing gene co-expression network to identify the hub genes closely related to PTC. In order to better explain the biological function genes, gene set enrichment analysis (GSEA) on hub genes was performed. GSEA can assess whether an a priori defined set of genes shows statistically significant, concordant differences between two biological states [19]. Furthermore, we analyzed the relationship between hub genes and tumor immune infiltrating cells since microenvironment composed of tumor immune infiltrating cells can play an important role in the occurrence and progression of tumor by promoting tumor and anti-tumor [20], and the influence of different infiltrating immune cells on tumor is different [21]. Our study might provide some new insights into current diagnosis and pathogenesis for PTC.

## Methods

### Thyroid cancer gene expression datasets collection and identification of robust DEGs

The data sets enrolled in this study needs to meet the main conditions [1]: The data set must include the gene expression profile of PTC and normal thyroid tissue [2]. The genes in the platform need to be above 5000. The 6 unprocessed gene expression profiles that met the inclusion criteria of this study were downloaded from the Gene Expression Omnibus (GEO, http://www.ncbi.nlm.nih.gov/geo/) database: GSE6004 [22], GSE58545 [23], GSE27155 [24, 25], GSE53157 [26], GSE60542 [27] and GSE33630 [28, 29]. The relevant data are shown in Table 1. For more detailed sample information, please see Additional file 2: Table S1. After data normalization using robust multi-array averaging algorithm [30], “limma” [31] package were used to assess statistical significance (P-value) for each gene based on a linear model implemented. Then, gene lists in six datasets were integrated using the RRA method to identify differentially expressed genes (DEGs) according to P-value in “RobustRankAggreg” package of R software, and P-value < 0.05 was set as the threshold for DEGs.

**Table 1:** Characteristics of the data sets enrolled in the study

Dataset ID	Number of normal samples	Number of tumor samples	Country	GPL ID	Number of rows per platform
GSE6004	4	7	USA	GPL570	54,675
GSE58545	18	27	Poland	GPL96	22,283
GSE27155	4	51	USA	GPL96	22,283
GSE53157	2	7	Portugal	GPL570	54,675
GSE60542	26	28	Belgium	GPL570	54,675
GSE33630	45	49	Belgium	GPL570	54,675

### Function enrichment analysis

The top 300 DEGs were uploaded to Database for Annotation, Visualization, and Integrated Discovery (DAVID) [32] for Gene Ontology (GO) functional annotation and Kyoto Encyclopedia of Genes and Genomes (KEGG) pathway enrichment analysis. The top terms were visualized in “GOplot” [33] of R software.

### Weighted co-expression network construction and key modules identification

1807 DEGs with P < 0.05 obtained from RRA to construct gene co-expression networks with expression data retrieved from The Cancer Genome Atlas (TCGA). Specifically, 1807 genes were selected from the TCGA gene list, and then the 1807 genes with their gene expression values and TCGA sample Numbers formed a correlation matrix. The threshold value of outliers was Z.K value < − 2.5. The correlation matrix (S_ij_) was converted to an adjacency matrix (A_ij_) based on soft threshold β that can approximate the scale-free distribution (R^2^ > 0.8). This transformation allows us to build networks with higher biological signals, which is the focus of the WGCNA approach. Topological overlap matrix (TOM) realizes the visualization of network, which is a simplified network diagram for identifying modules. The hierarchical clustering tree formed by average linkage hierarchical clustering also participates in the formation of modules. By the way, some genes without characteristics were assigned to the gray module. In order to identify the key modules closely related to PTC, module eigengene (ME), gene significance (GS), module membership (MM) and other parameters were calculated [34]. The genes in the key modules were uploaded to DAVID [32] for GO functional annotation and KEGG enrichment analysis to explore the biological functions of the key modules.

### Identification, validation and efficacy evaluation for hub genes

The hub gene is defined as the gene with the highest degree of connectivity in the key module. Specifically, the genes with geneModuleMembership > 0.9 and geneTraitSignificance > 0.5 were determined as the hub genes in the study. Samples in the Cancer Genome Atlas-Thyroid carcinoma (TCGA-THCA) and a separate data set GSE29265 was used to verify that hub genes can distinguish between non-tumor and PTC. If the P-value < 0.01, the selection of the hub gene is considered statistically significant. In addition, we conducted a study to understand the expression patterns of hub genes between different stages of PTC based on GEPIA (Gene Expression Profiling Interactive Analysis, http://gepia.cancer-pku.cn/) [35], which was a web server for cancer and normal gene expression profiling and interactive analyses. To assess diagnostic values of hub genes, receiver operating characteristic (ROC) curve was plotted and area under the ROC curve (AUC) was calculated with “pROC” of R package [36] to evaluate the capability of distinguishing tumor and normal tissue.

### Methylation analysis of hub genes

Methylation is one of the earliest and most widely studied epigenetics to be included in our study. We conducted methylation analysis of hub genes based on methylation data obtained from the human disease methylation database version 2.0 [37, 38] (DiseaseMeth 2.0, http://bioinfo.hrbmu.edu.cn/diseasemeth/), which collects and annotates the abnormal methylation of various cancers, is a useful resource platform for further understanding the molecular mechanisms of human disease. Data in the platform based on huge international disease projects including TCGA and public genome databases including GEO [37]. Furthermore, the relationship between hub genes expression and their DNA methylation status were investigated based on MEXPRESS (http://mexpress.be) [39].

### Hub genes and tumor-infiltrating immune cells

Due to tumor immune infiltrating cells (B cells, CD4^+^ T cells, CD8^+^ T cells, neutrophils, macrophages, and dendritic cells) are closely related to prognosis, thus it is necessary to investigate the correlation between the expression of identified hub genes and tumor infiltrating immune cells, which might provide new ideas for immunotherapy. This analysis for immune infiltrating cells and their interactions with tumor cells [40, 41] was performed on the basis of TIMER, which use to comprehensively investigate molecular characterization of tumor-immune interactions.

### Gene set enrichment analysis

GSEA, which is widely used to predict the biological functions of hub genes, were performed in GSEA 4.0.3 and the top terms were visualized in R. The samples in TCGA were divided into two groups (high expression vs. low expression) with median expression values of each hub gene.

## Results

### Identification of robust DEGs

The P-value of each gene in 6 datasets was calculated based on the “limma” package. Gene lists of six datasets were integrated by RRA method and 1807 DEGs including 796 up-regulated genes and 1011 down-regulated genes were identified to the threshold of P-value < 0.05. The top 20 down-regulated and up-regulated genes were listed based on heat maps (Additional file 1: Figure S1).

### Function enrichment analysis

**Fig. 1:**
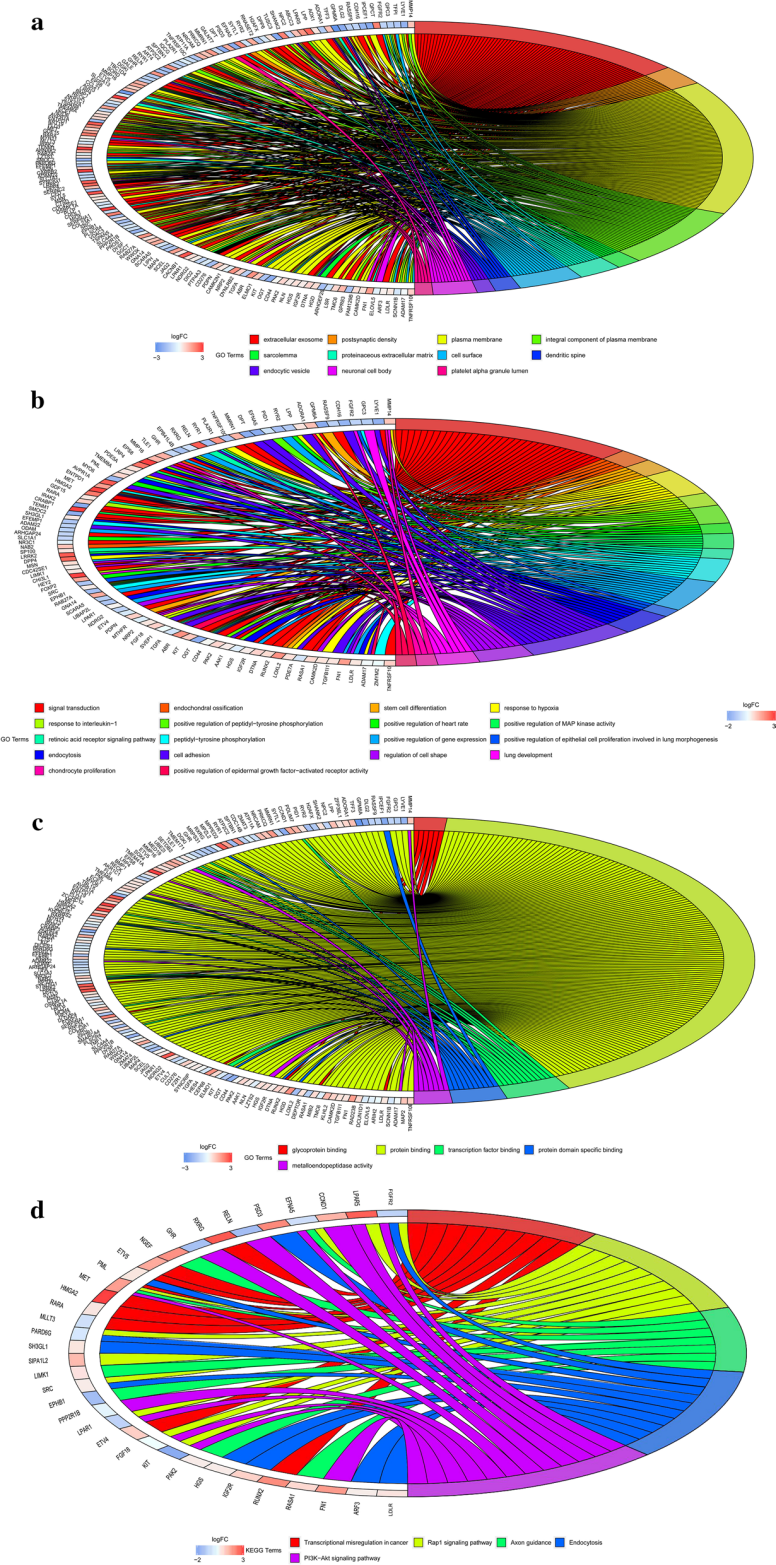
Chord diagrams for GO and KEGG analysis of top 300 DEGs. The link between genes and pathways was described grounded on GO cellular components. **a** The link between genes and pathways was described grounded on GO biological processes. **b** The link between genes and pathways was described grounded on GO molecular function. **c** The link between genes and pathways was described grounded on KEGG. **d** Different genes and pathways are color-coded according to the catalog

Chord diagrams showed the biological pathways in which the top 300 genes were involved. The top terms were illustrated in Fig. 1. Figure 1a shows that these genes are enriched into extracellular exosome, plasma membrane, integral component of plasma membrane etc. based on GO cellular components. Figure 1b shows that these genes are enriched into signal transduction, response to interleukin − 1, stem cell differentiation and positive regulation of MAP kinase activity etc. based on GO biological processes. Figure 1c shows that these genes are enriched to glycoprotein binding, protein binding and metalloendopeptidase activity etc. based on GO molecular function. These genes were enriched into the transcriptional misregulation in cancer, Rap1 signaling pathway and PI3K − Akt signaling pathway etc. based on KEGG (Fig. 1d).

### Weighted co-expression network construction and key modules identification

**Fig. 2:**
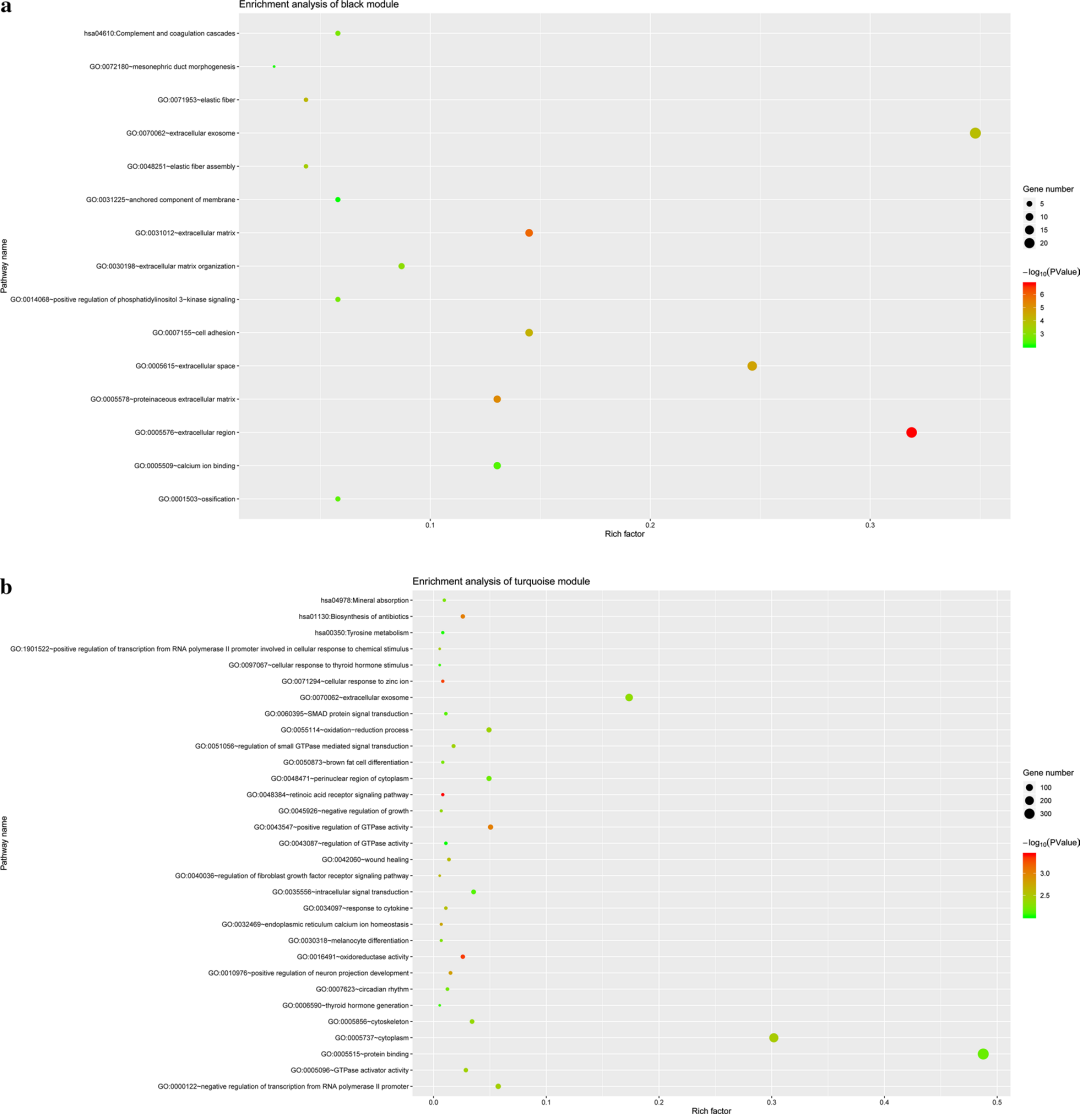
Enrichment analysis of black and turquoise modules based on GO and KEGG. **a** Indicates enrichment analysis of black module, and **b** indicates enrichment analysis of turquoise module. The pathways (vertical axis) and rich factor (horizontal axis) were shown, and the size and color of nodes that represent genes were depicted according to the legends

Since there were 5 samples with Z.K value < − 2.5 (TCGA.KS.A41F, TCGA.EM.A3O3, TCGA.DJ.A2Q1, TCGA.DE.A4MA and TCGA.BJ.A3F0), these 5 samples were considered as outliers and excluded from the subsequent analysis (Additional file 1: Figure S2). Matrix transformation is based on soft threshold β = 7 (scale free R^2^ = 0.88) (Additional file 1: Figure S3), which is selected according to scale-free topological criteria. 10 gene modules were identified based on TOM and average linkage hierarchical clustering (Additional file 1: Figure S4). In order to identify the modules associated with clinical characteristics, the ME that represents the gene expression profile of each module was calculated. The MEblack (r = − 0.64, P = 5e − 38) and MEturquoise (r = − 0.65, P = 3e − 39) were considered to be the key modules most associated with thyroid cancer (Additional file 1: Figure S5). At the same time, the module membership vs. gene significance showed that the black and turquoise modules were closely related to the disease (Additional file 1: Figure S6). GO and KEGG indicated that black module (Fig. 2a) was mainly enriched to extracellular region, extracellular matrix and extracellular space etc. GO and KEGG indicated that turquoise module was (Fig. 2b) mainly enriched to retinoic acid receptor signaling pathway, oxidoreductase activity and cellular response to zinc ion etc.

### Identification, validation and efficacy evaluation for hub genes

**Fig. 3:**
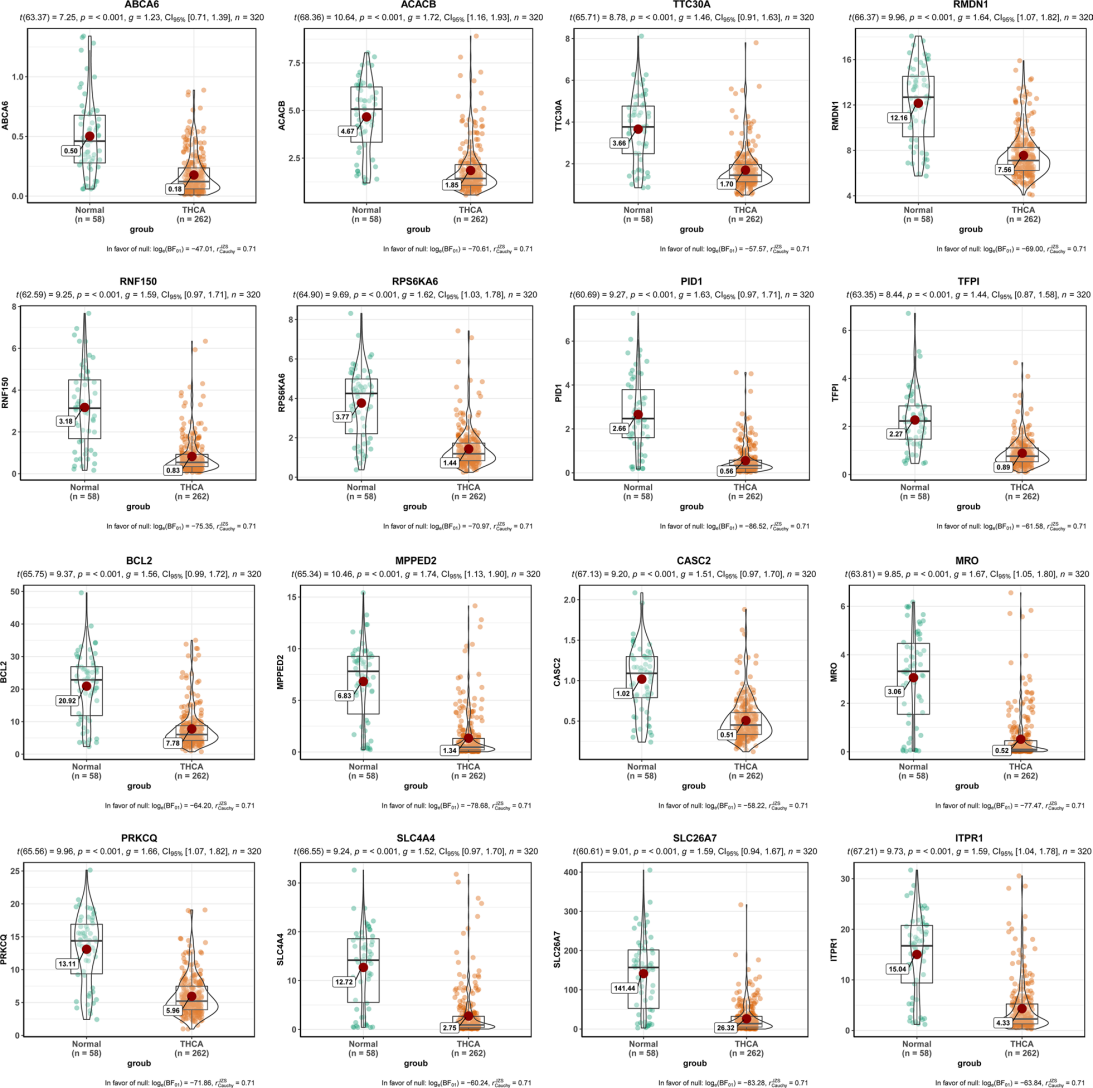
Sixteen hub genes were verified based on TCGA. The 16 hub genes were significantly down-regulated

**Fig. 4:**
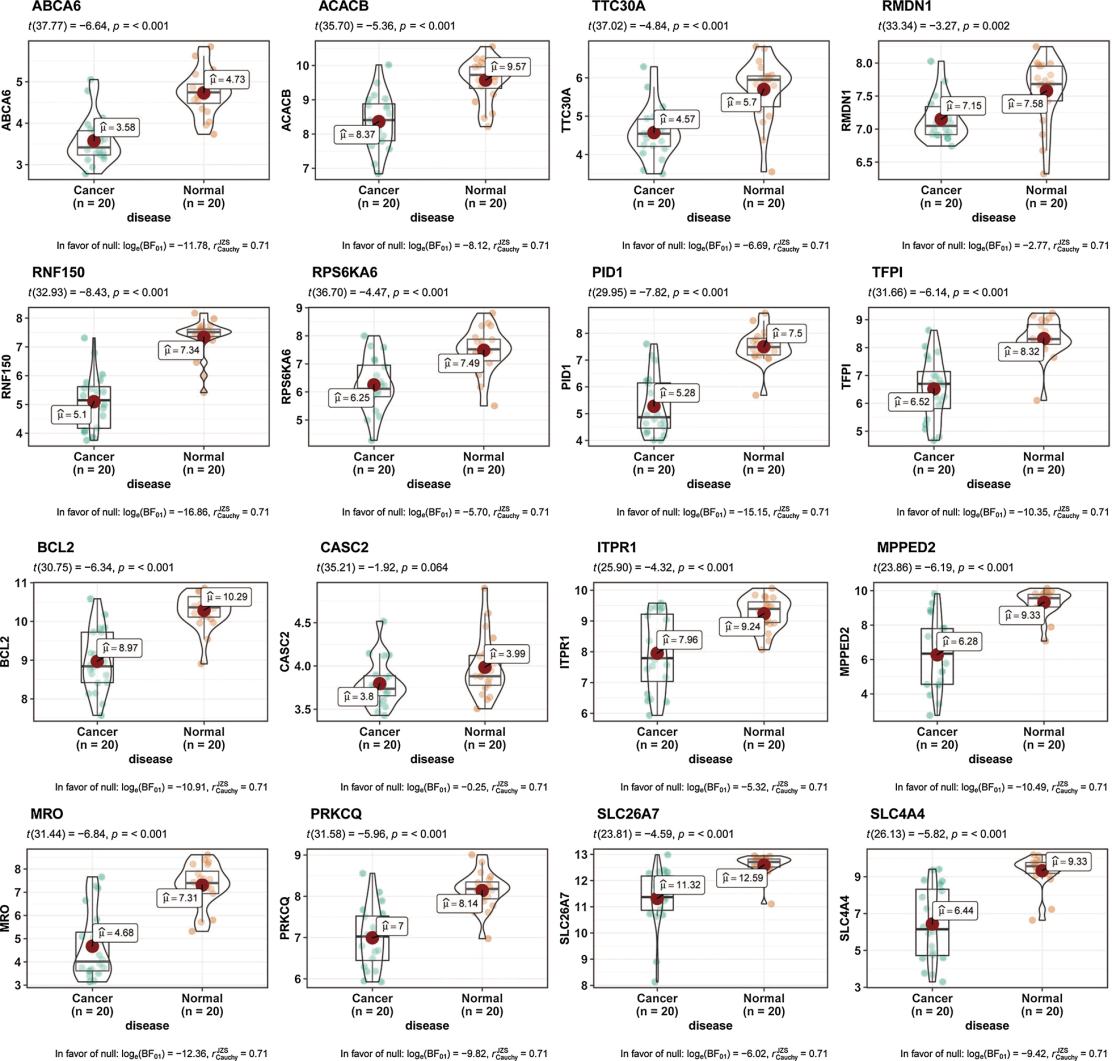
Sixteen hub genes were verified based on GSE29265. The 16 hub genes were significantly down-regulated

**Fig. 5:**
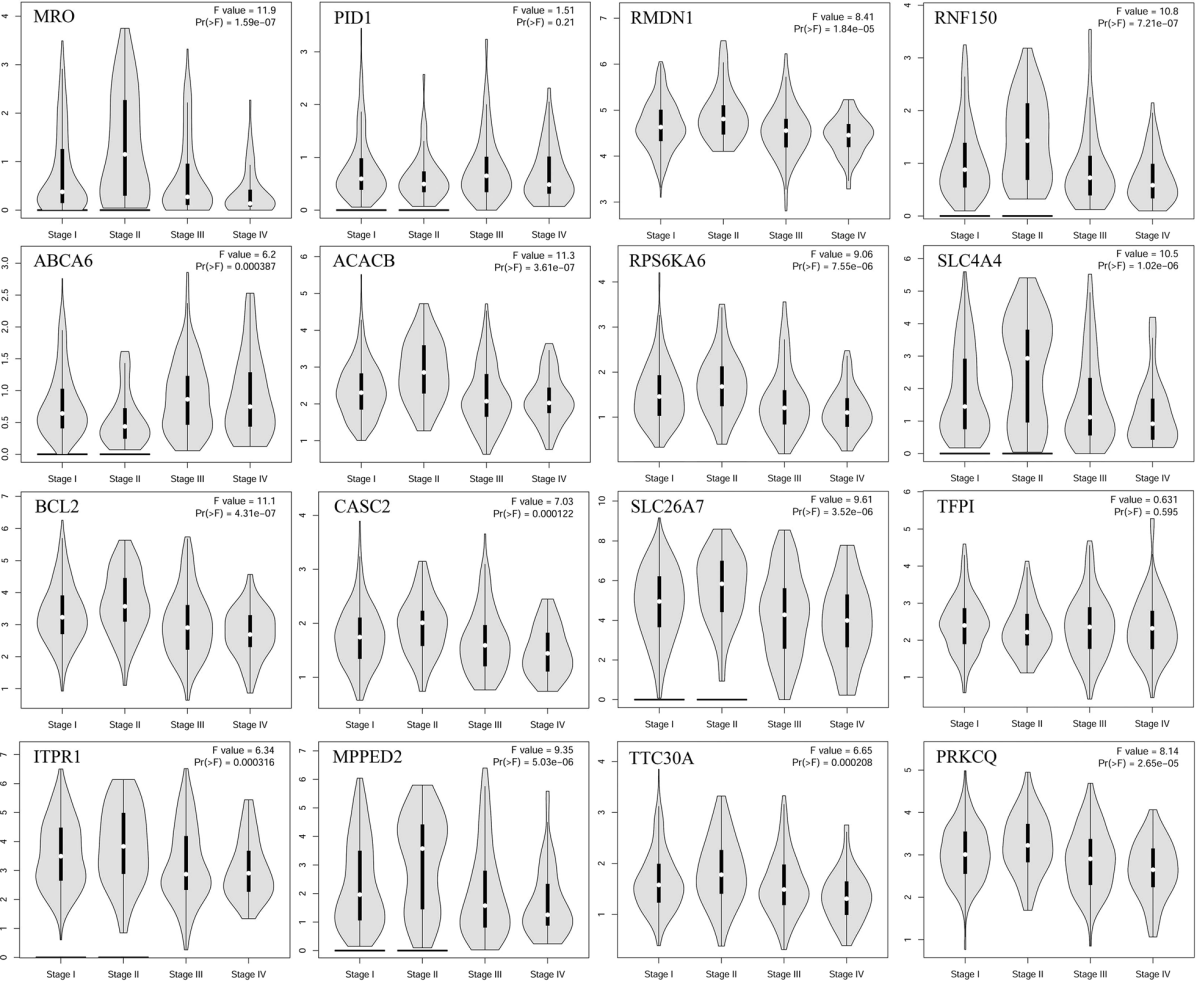
Pathological stage plot of THCA from GEPIA

16 genes with geneModuleMembership > 0.9 and geneTraitSignificance > 0.5 were determined as the hub genes in Table 2. Figure 3 (TCGA-THCA) and Fig. 4 (GSE29265) intuitively showed that 16 hub genes are significantly down-regulated. Figure 5 ploted in GEPIA illustrated that there were some differences in expression patterns in different stages of PTC. The main difference was between stage I, II, and III: the expression patterns of ABCA6, PID1and TFPI were down in stage I–II–up in stage II–III, ACACB, BCL2, CASC2, ITPR1, MPPED2, MRO, PRKCQ, RMDN1, RNF150, RPS6KA6,SLC26A7, SLC4A4 and TTC30A were up in stage I–II–down in stage II–III (Table 2). Furthermore, ROC curves showed high diagnostic value of 16 hub genes for PTC (Additional file 1: Figure S7A, B): except for ABCA6 AUC = 89.5%, the 15 genes with AUC > 90%. To our knowledge, the eight hub genes (ABCA6, ACACB, TTC30A, RMDN1, RNF150, RPS6KA6, PID1 and TFPI) in Fig. 3 have been poorly studied. Therefore, we focused on these 8 hub genes in this study.

**Table 2:** The list of sixteen hub genes identified in gene expression network

Gene symbol	GeneModuleMembership	GeneTraitsignificance	Expression pattern
PID1	0.915896493	0.66001985	Down in stage I–II–up in stage II–III
ABCA6	0.911773582	0.524366279	Down in stage I–II–up in stage II–III
TFPI	0.903220611	0.599708264	Down in stage I–II–up in stage II–III
MPPED2	0.945621212	0.637198678	Up in stage I–II–down in stage II–III
RPS6KA6	0.932048783	0.611335416	Up in stage I–II–down in stage II–III
MRO	0.931567859	0.632361438	Up in stage I–II–down in stage II–III
RMDN1	0.923154211	0.610969324	Up in stage I–II–down in stage II–III
ACACB	0.919880602	0.610967988	Up in stage I–II–down in stage II–III
SLC4A4	0.913670371	0.575022712	Up in stage I–II–down in stage II–III
TTC30A	0.912368213	0.563286233	Up in stage I–II–down in stage II–III
RNF150	0.909497328	0.625793803	Up in stage I–II–down in stage II–III
BCL2	0.909385573	0.588759014	Up in stage I–II–down in stage II–III
CASC2	0.908891219	0.567289908	Up in stage I–II–down in stage II–III
PRKCQ	0.907179413	0.615140746	Up in stage I–II–down in stage II–III
SLC26A7	0.903842658	0.648569813	Up in stage I–II–down in stage II–III
ITPR1	0.900631876	0.587725656	Up in stage I–II–down in stage II–III

GeneModuleMembership, GeneTraitSignificance and expression pattern of hub genes were showed. The cut-off thresholds of hub genes were geneModuleMembership > 0.9 and geneTraitSignificance > 0.5, and all hub genes are significantly down-regulated

### Methylation analysis of hub genes

As far as we know, DNA methylation shuts down the activity of some genes and demethylation induces gene expression. Additional file 1: Figure S8 shown that ABCA6, ACACB, RMDN1 and TFPI were significantly different (P < 0.05), so ABCA6, ACACB, RMDN1 and TFPI were defined as differentially methylated genes (DMGs). Moreover, the Additional file 1: Figure S9A–D illustrated that the expression levels of 4 DMGs are negatively correlated with DNA methylation in MEXPRESS. That indicate the abnormal down-regulation of DMGs likely resulted from hypermethylation.

### Hub genes and tumor-infiltrating immune cells

Immune cells play an important role in the development and progression of tumors. Immunotherapy for cancer is becoming more and more important, so it is necessary to study the relationship between hub genes and immune cell infiltration. We studied the relationship between 8 hub genes and different immune cells based on TIMER. However, RMDN1was not involved in this analysis since hub genes RMDN1 is not recognized by TIMER. The seven hub genes were all correlated with the immune cells (B cells, CD4^+^ T cells, CD8^+^ T cells, neutrophils, macrophages, and dendritic cells) and tumor purity in the study (Additional file 1: Figure S10A–G).

### Gene set enrichment analysis

**Fig. 6:**
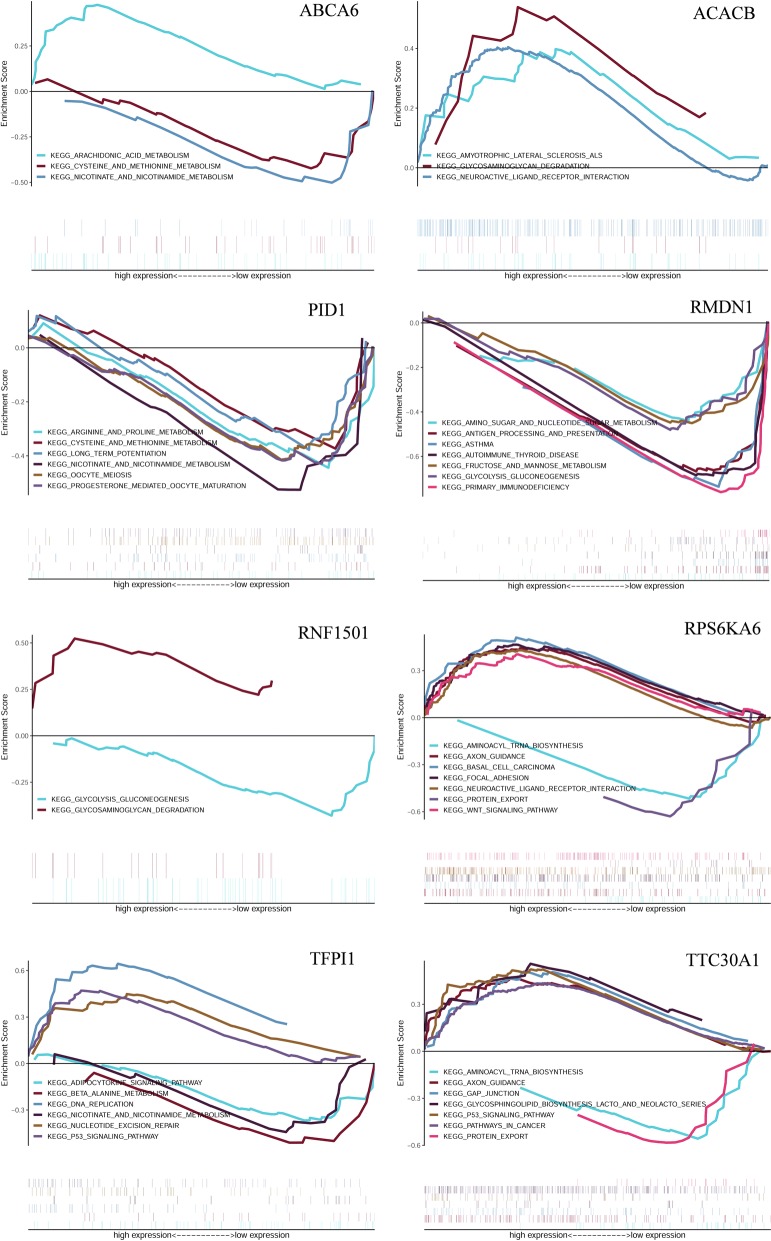
Gene set enrichment analysis for hub gene

In order to further explore the expression pathways of 8 hub genes, we conducted GSEA, which is widely used to predict the biological functions of hub genes. These results suggest that these genes are associated with PTC (Fig. 6). ABCA6, PID1, RMDN1, RPS6KA6, TTC30A and TFPI were involved in amino acid metabolism. ACACB, RMDN1, RNF150, TTC30A have been shown to be involved in carbohydrate anabolism. PID1, TFPI and ABCA6 may be involved in the niacin and niacinamide metabolism.

## Discussion

The information of 6 data sets was integrated based on RRA, and 1807 DEGs were screened as the threshold of P-value < 0.05. In order to explore the specific biological role of these genes, the top 300 DEGs were analyzed by GO functional annotation and KEGG enrichment based on DAVID. Functional annotation of the top 300 DEGs may be helpful to the underlying mechanism of PTC. GO terms were obtained such as signal transduction, response to interleukin-1, positive regulation of MAP kinase activity, extracellular exosome, plasma membrane, integral component of plasma membrane and metalloendopeptidase activity. Signal transduction is very important in the occurrence of tumor [42]. Interleukin-1 plays a role in regulating innate immunity and adaptive immunity [43]. In particular, interleukin-1 has dual roles of anti-tumor and pro-tumor [43]. Positive regulation of MAP kinase activity and metalloendopeptidase activity are thought to be associated with thyroid cancer [44, 45]. This is a validation of the major carcinogenic event of PTC is the activation of MAPK. Meanwhile, KEGG terms such as transcriptional misregulation in cancer, Rap1 signaling pathway and PI3K-Akt signaling pathway were obtianed. PI3K-Akt signaling pathway had been reported to play an important role in the development of thyroid cancer [46, 47], breast cancer [48], colorectal cancer [49], non-small cell lung cancer [50] and gastric cancer [51] etc.

In order to identify the hub genes in the 1807 DEGs, 1807 genes were used to construct a gene co-expression network. MEblack and MEturquoise showed high correlations with PTC. Enrichment analysis showed that the genes in the black module were mainly related to extracellular matrix, and the genes in the turquoise module were enriched to retinoic acid receptor signaling pathway, oxidoreductase activity and cellular response to zinc ion etc. As the main bioactive metabolite of vitamin A, retinoic acid plays a regulatory role in proliferation and differentiation [52], and it has a profound impact on adipogenesis by activating retinoic acid receptors [53]. It is important to note that retinoic acid receptor signaling pathway may play a role in immune suppression. Previous studies have shown that abnormalities in the retinoic acid receptor signaling pathway are associated with various malignancies [54], but the specific mechanism between this pathway and PTC remains unclear. We identified 16 hub genes based on our selection criteria and ROC curve indicated that 16 hub genes had good diagnostic value, 8 of which were focused on. Not only did these genes differ significantly between tumours and non-tumours, but their expression patterns differed at different stages based on validation for hub genes. Methylation analysis showed that ABCA6, ACACB, RMDN1 and TFPI were identified as DMGs, and significant down-regulation of DMGs in patients with PTC might be realized by the hypermethylation. DNA methylation is reversible, so targeted therapies are attractive in cancer. The combination of genetic alterations and DNA methylation alterations may improve their clinical value. Therefore, 16 hub genes and DNA methylation of DMGs as potential biomarkers may be used to diagnose state (inert or invasive) of PTC for selecting the most appropriate treatment plan at present, so as to avoid overtreatment, improve the diagnosis of invasive tumor. This may provide new insights into refractory radioiodine differentiated tumors. The expression levels of 8 hub genes were significantly correlated with tumor purity, and these genes were moderately correlated with tumor-infiltrating immune cells. Furthermore, GSEA for hub genes predicted biological functions of these genes. ABCA6 belongs to the superfamily of ATP-binding cassette transporters and ABCA6 may be related to lipid homeostasis in macrophages [55]. Although some members of the superfamily of ATP-binding cassette transporters play important roles in tumor-generating mechanisms and drug resistance [56], the specific biological role of ABCA6 is not clearly understood. ACACB has been reported in laryngeal squamous cell carcinoma [57], nasopharyngeal carcinoma [58] and hepatocellular carcinoma [59]. PID1, a gene that regulates the sensitivity of fat and muscle cells to insulin signals, has been reported as a feature gene in several cancers, including thyroid cancer [60]. RPS6KA6, also known as RSK4, is considered as a tumor suppressor gene due to its resistance to invasion and metastasis, which may be related with inhibition of the MAPK pathway [61]. The activation of MAPK likely resulted from down-regulated of RPS6KA6. RPS6KA6 has been significantly down-regulated in colorectal cancer [62], ovarian cancer [63], non-small cell lung cancer [64], breast cancer, acute myeloid leukemia [65], etc., while this study is the first to mention that RPS6KA6 is significantly down-regulated in PTC. By the way, RPS6KA6 is also considered a DMG in esophageal cancer [66]. It is worth mentioning that RPS6KA6 was considered as a drug resistance marker for the treatment of cancer by protein kinase inhibitors in a study in 2012 [67]. In general, there has been little research on these 8 hub genes, especially RMDN1, TTC30A and RNF150, thus, it is imperative to fully uncover the specific relationship between hub genes and PTC.

Although WGCNA is widely used as a powerful data-driven tool for various diseases including various solid malignancies and hematologic malignancies, there is little research on establishing gene co-expression networks to identify genes that play a pivotal role in PTC. A new study using the WGCNA method to identify hub genes suggests that 11 hub genes may be involved in the recurrence of papillary thyroid cancer [68]. A total of 16 hub genes were identified in this study, and these 16 genes did not overlap with the above 11 genes. In particular, our study is the first to study PTC using a combination of RRA and WGCNA. However, we cannot deny that there may be bias due to the small sample sizes, more studies are needed to validate our results.

## Conclusions

In summary, we identified 1807 DEGs from 6 datasets of PTC data based on RRA methods. From the gene co-expression network, we identified 16 hub genes, which were shown to be significantly down-regulated and expression patterns differed at different stages according to hub gene verification. ROC curve indicated that 16 hub genes had good diagnostic value. For methylation analysis, ABCA6, ACACB, RMDN1 and TFPI were identified as DMGs, and MEXPRESS indicated that decreased expression level of these genes may relate to abnormal DNA methylation. Hub genes and methylation of DMGs may as potential biomarkers provide new insights for diagnosis of PTC. The expression levels of 8 hub genes were correlated with tumor purity as well as tumor-infiltrating immune cells. Although these hub genes were found in PTC, the specific role of these hub genes in the underlying mechanism of PTC is not clear. Therefore, GSEA provides insights into the biological functions of hub genes, which might be the direction of future work. In addition, functional annotations for the top300 DEGs and key modules provide insight into the underlying mechanism of PTC. To further understand the pathogenesis of PTC is the key to adjust the current diagnosis, which can further improve the prognosis of PTC patients.

## Supplementary information

**Additional file 1: Figure S1.** Heat map for top 20 down-regulated and top 20 up-regulated genes. **Figure S2.** Sample dendrogram and trait heatmap. **Figure S3.** Analysis of network topology for various soft-thresholding powers. **Figure S4.** Cluster dendrogram. Modules identified by WGCNA based on a dissimilarity measure (1-TOM). **Figure S5.** Heat map for the correlation between ME and clinical traits of thyroid cancer. Each gene module was colored according to legend. **Figure S6.** Scatter diagram for black and turquoise modules. **Figure S7A-B.** ROC curves for hub genes. **Figure S8.** Boxplot for methylation analysis of 8 hub genes. **Figure S9A-D.** Association of Methylation sites with expression of 4 differentially methylated genes (DMGs). **Figure S10.** The relationship between hub gene expression and tumor purity and tumor infiltrating immune cells.

**Additional file 2: Table S1.** Details of the six databases.

## Data Availability

The data that support the findings of this study are openly available in GEO (https://www.ncbi.nlm.nih.gov/geo/) and TCGA (https://www.cancer.gov/about-nci/organization/ccg/research/structuralgenomics/tcga).

## Abbreviations

PTC: Papillary thyroid cancer,
MAPK: Mitogen-activated protein kinase,
anti-CTLA-4: Anti-cytotoxic T lymphocyte antigen 4,
anti-PD-1/PD-L1: Anti-programmed cell death protein-1/programmed cell death ligand-1
BRAF: B-Raf proto-oncogene, serine/threonine kinase
DAVID: Database for annotation, visualization, and integrated discovery
DEGs: Differentially expressed genes
ROC: Receiver operating characteristic
AUC: Area under the ROC curve
DMGs: Differentially methylated genes,
GEPIA: Gene expression profiling interactive analysis
GEO: Gene expression omnibus
GO: Gene ontology
GS: Gene significance
GSEA: Gene set enrichment analysis
KEGG: Kyoto encyclopedia of genes and genomes
ME: Module eigengene
MM: Module membership
RRA: Robust rank aggregation
TCGA: The cancer genome atlas
TCGA-THCA: The cancer genome atlas-thyroid carcinoma
TOM: Topological overlap matrix
WGCNA: Weighted gene co-expression network analysis
