# Supplementary material for: Identification of hub genes in papillary thyroid carcinoma: robust rank aggregation and weighted gene co-expression network analysis
Source: J Transl Med. 2020 Apr 16;18:170. doi: 10.1186/s12967-020-02327-7 (PMC7161219; doi:10.1186/s12967-020-02327-7)
Supplement: Supplementary file 1 — Additional file 1: Figure S1. Heat map for top 20 down-regulated and top 20 up-regulated genes. Figure S2. Sample dendrogram and trait heatmap. Figure S3. Analysis of network topology for various soft-thresholding powers. Figure S4. Cluster dendrogram. Modules identified by WGCNA based on a dissimilarity measure (1-TOM). Figure S5. Heat map for the correlation between ME and clinical traits of thyroid cancer. Each gene module was colored according to legend. Figure S6. Scatter diagram for black and turquoise modules. Figure S7A-B. ROC curves for hub genes. Figure S8. Boxplot for methylation analysis of 8 hub genes. Figure S9A-D. Association of Methylation sites with expression of 4 differentially methylated genes (DMGs). Figure S10. The relationship between hub gene expression and tumor purity and tumor infiltrating immune cells. [file 12967_2020_2327_MOESM1_ESM.docx]

**Additional file**

**Additional file Figure S1** Heat map for top 20 down-regulated and top 20 up-regulated genes.

**Note:** Data set ID (x-axis) and 40 genes (y-axis) were displayed, and each gene was colored according to color key.

**Additional file Figure S2** Sample dendrogram and trait heatmap.

**Noye:** 5 samples with a Z.K value <-2.5 were considered as outliers and excluded from the subsequent analysis.

**Additional file Figure S3** Analysis of network topology for various soft-thresholding powers.

**Additional file Figure S4** Cluster dendrogram. Modules identified by WGCNA based on a dissimilarity measure (1-TOM).

**Additional file Figure S5** Heat map for the correlation between ME and clinical traits of thyroid cancer. Each gene module was colored according to legend.

**Additional file Figure S6** Scatter diagram for black and turquoise modules.

**Note:** The left is module membership vs. gene significance in black module (cor=0.8, P=1.6e-16), the right is module membership vs. gene significance in turquoise module (cor=0.83, P=1.6e-187).

**Additional file Figure S7A-B** ROC curves for hub genes.

**Note:** The area under the curve (AUC) is proportional to the diagnostic value of the gene.(A) ROC curves of eight less noticed hub genes; (B) ROC curves of eight hub genes that received more attention.

**Additional file FigureS 8** Boxplot for methylation analysis of 8 hub genes.

**Additional file Figure S9A-D** Association of Methylation sites with expression of 4 differentially methylated genes (DMGs).

**Note:**  The samples are sorted the genes expression levels and the samples without expression data were removed. On the right side, Pearson s correlation coefficients and P values from Wilcoxon rank-sum test for methylation sites and query gene expression are shown the expression of genes and DNA methylation are negatively correlated.

**Additional file Figure S10** The relationship between hub gene expression and tumor purity and tumor infiltrating immune cells.

**Note:** Each black node represents a sample, where A indicates ABCA6, B indicates ACACB, C indicates TTC30A, D indicates RPS6KA6, E indicates RNF150, F indicates PID1, and G indicates TFPI.

**Supplemental Figure 1** Heat map for top 20 down-regulated and top 20 up-regulated genes.

**
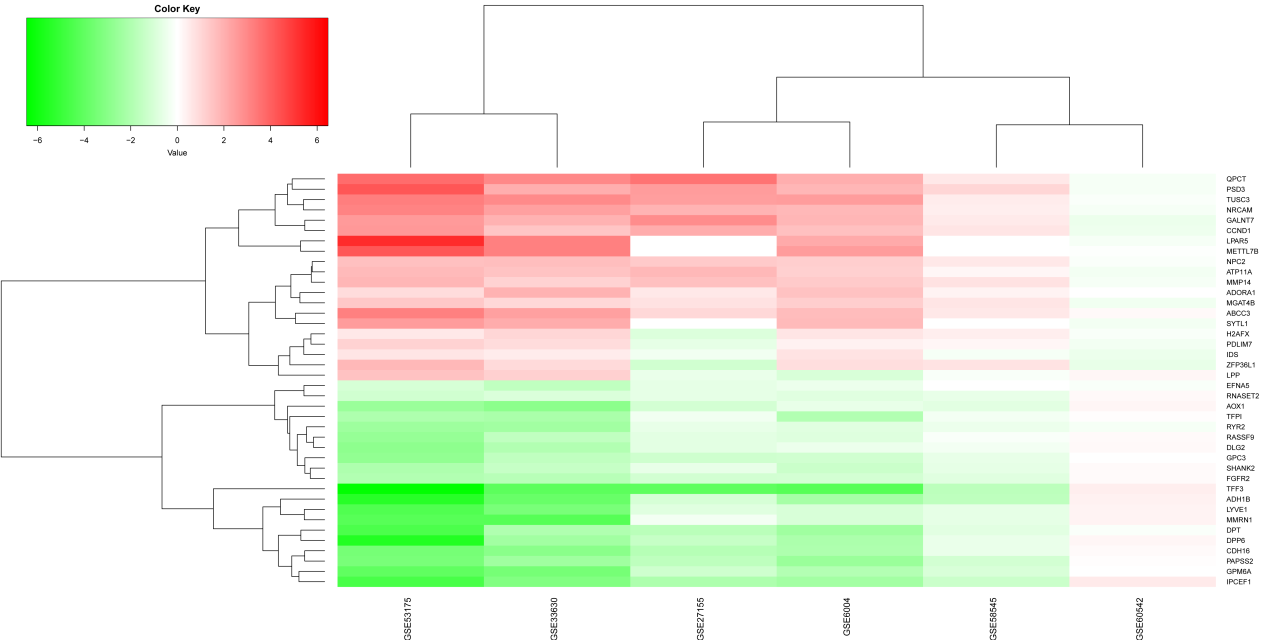
**

**Note:** Data set ID (x-axis) and 40 genes (y-axis) were displayed, and each gene was colored according to color key.

**Additional file Figure 2** Sample dendrogram and trait heatmap.


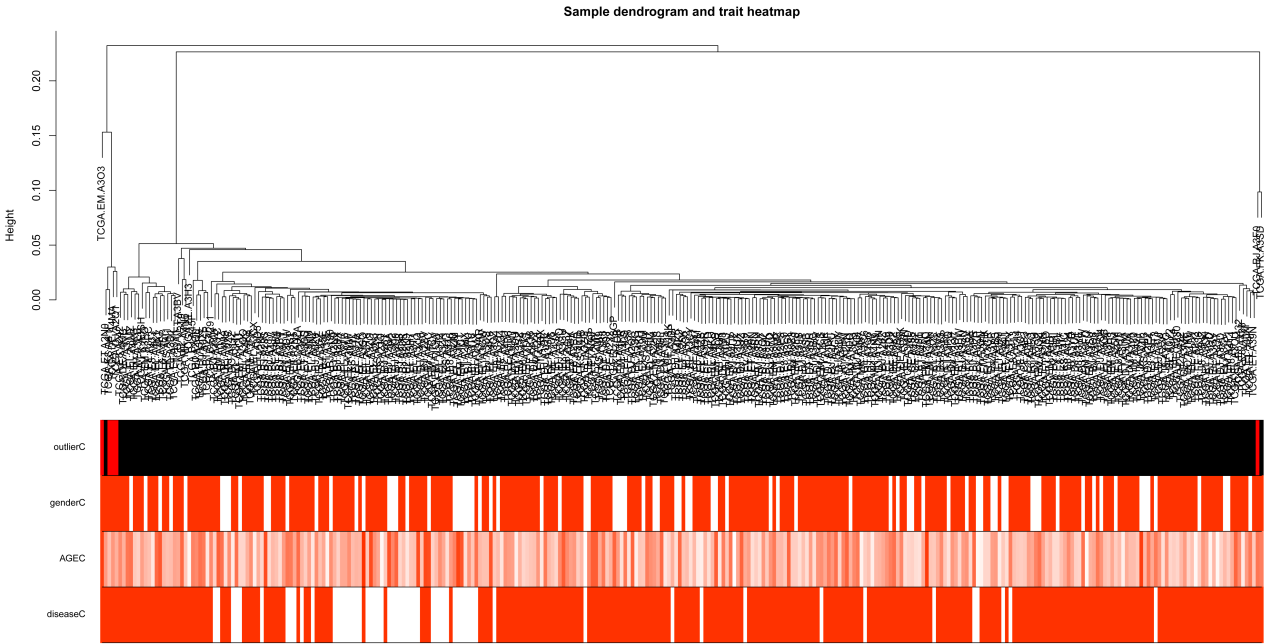


**Noye:** 5 samples with a Z.K value <-2.5 were considered as outliers and excluded from the subsequent analysis.

**Additional file Figure 3** Analysis of network topology for various soft-thresholding powers.


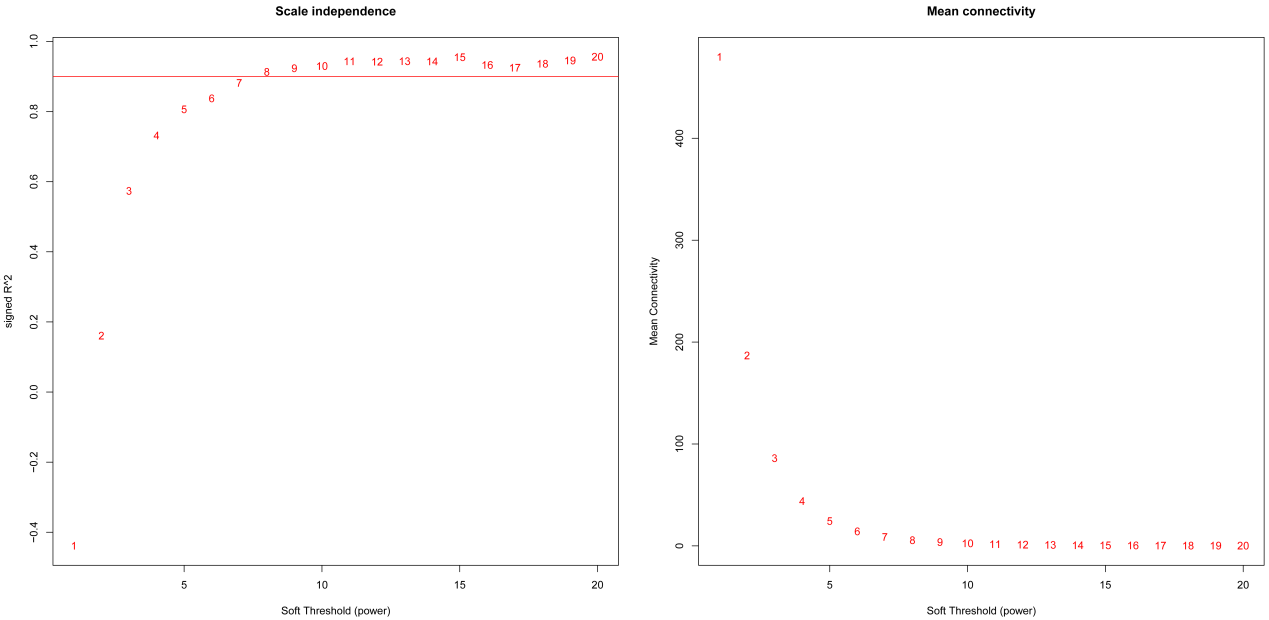


**Additional file Figure 4** Cluster dendrogram. Modules identified by WGCNA based on a dissimilarity measure (1-TOM).


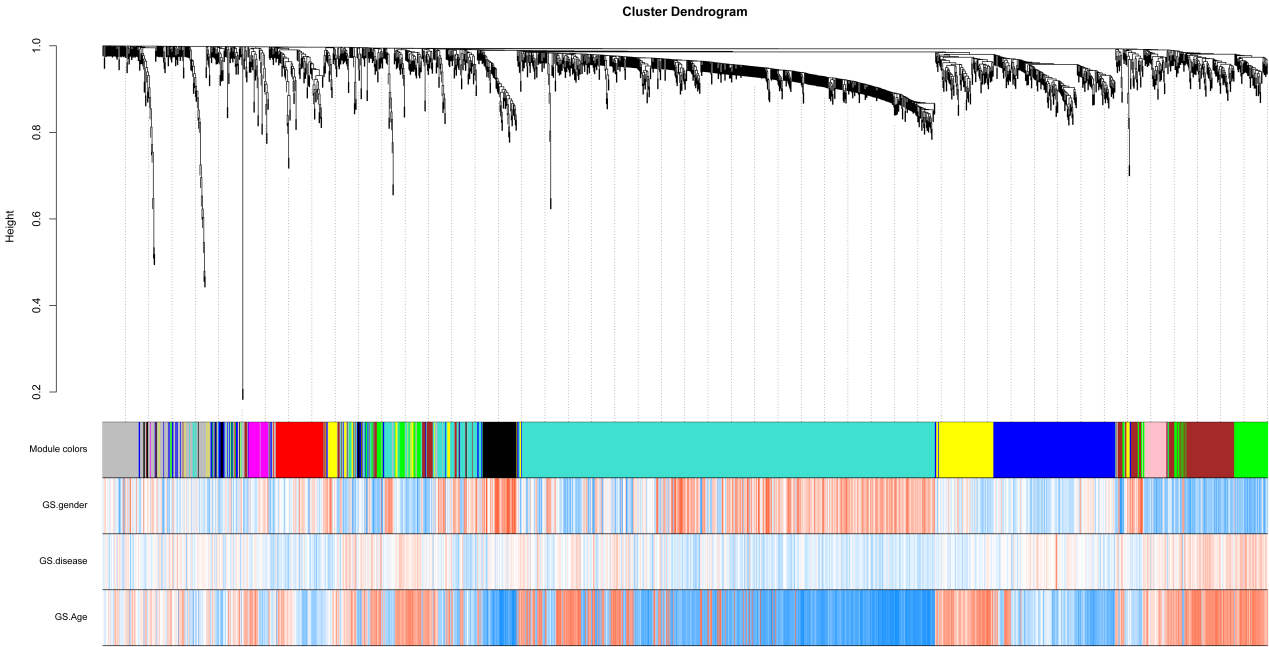


**Additional file Figure 5** Heat map for the correlation between ME and clinical traits of thyroid cancer. Each gene module was colored according to legend.


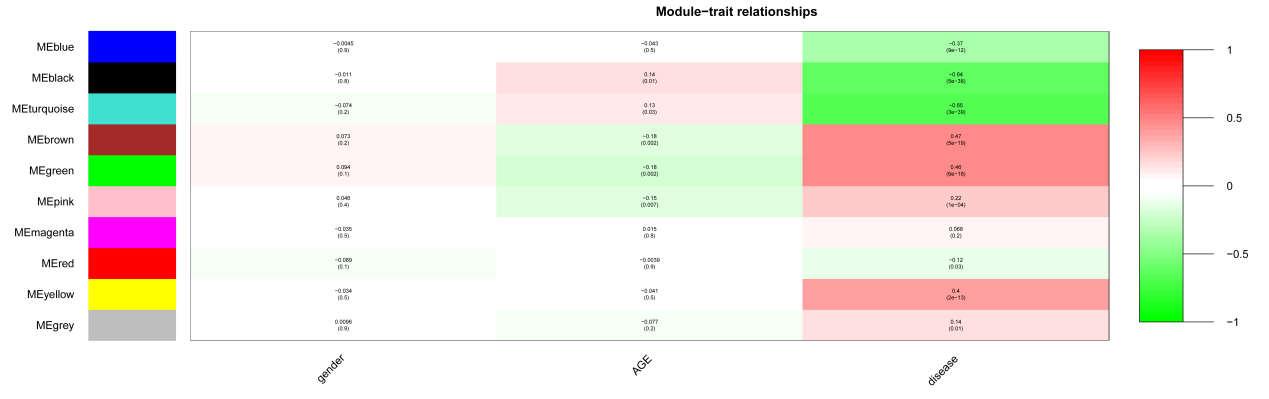


**Additional file Figure 6** Scatter diagram for black and turquoise modules.


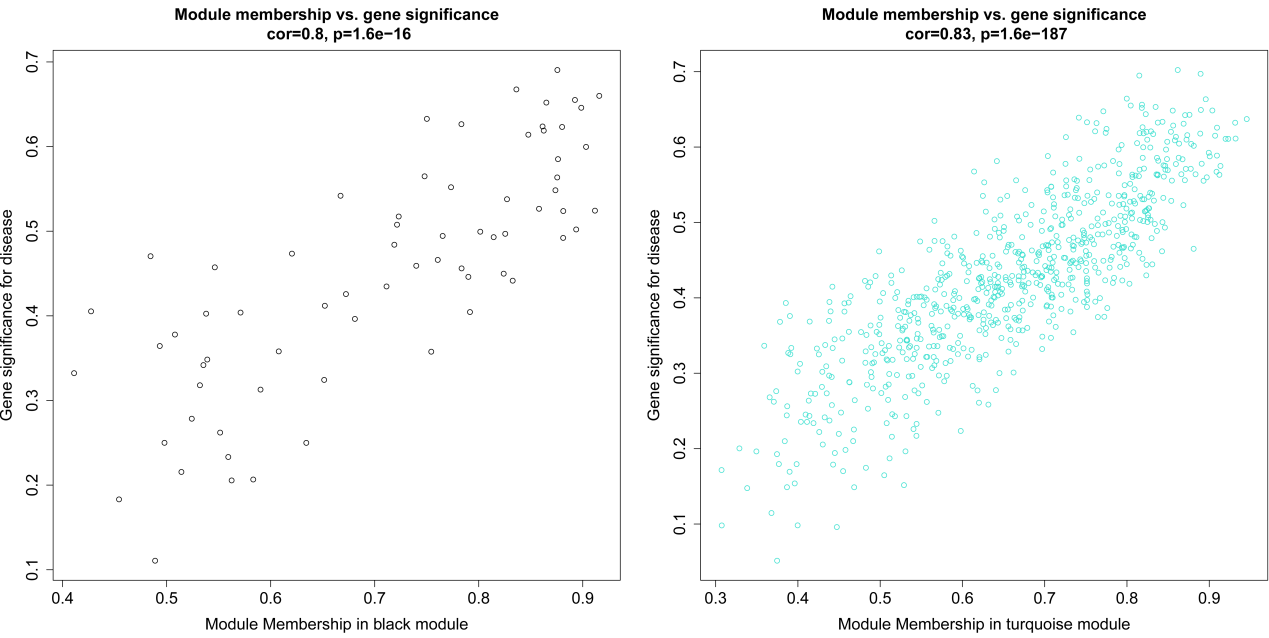


**Note:** The left is module membership vs. gene significance in black module (cor=0.8, P=1.6e-16), the right is module membership vs. gene significance in turquoise module (cor=0.83, P=1.6e-187).

**Supplementary Figure 7A-B** ROC curves for hub genes.

**Note:** The area under the curve (AUC) is proportional to the diagnostic value of the gene. (A) ROC curves of eight less noticed hub genes; (B) ROC curves of eight hub genes that received more attention.

7A ROC curves of eight less noticed hub genes


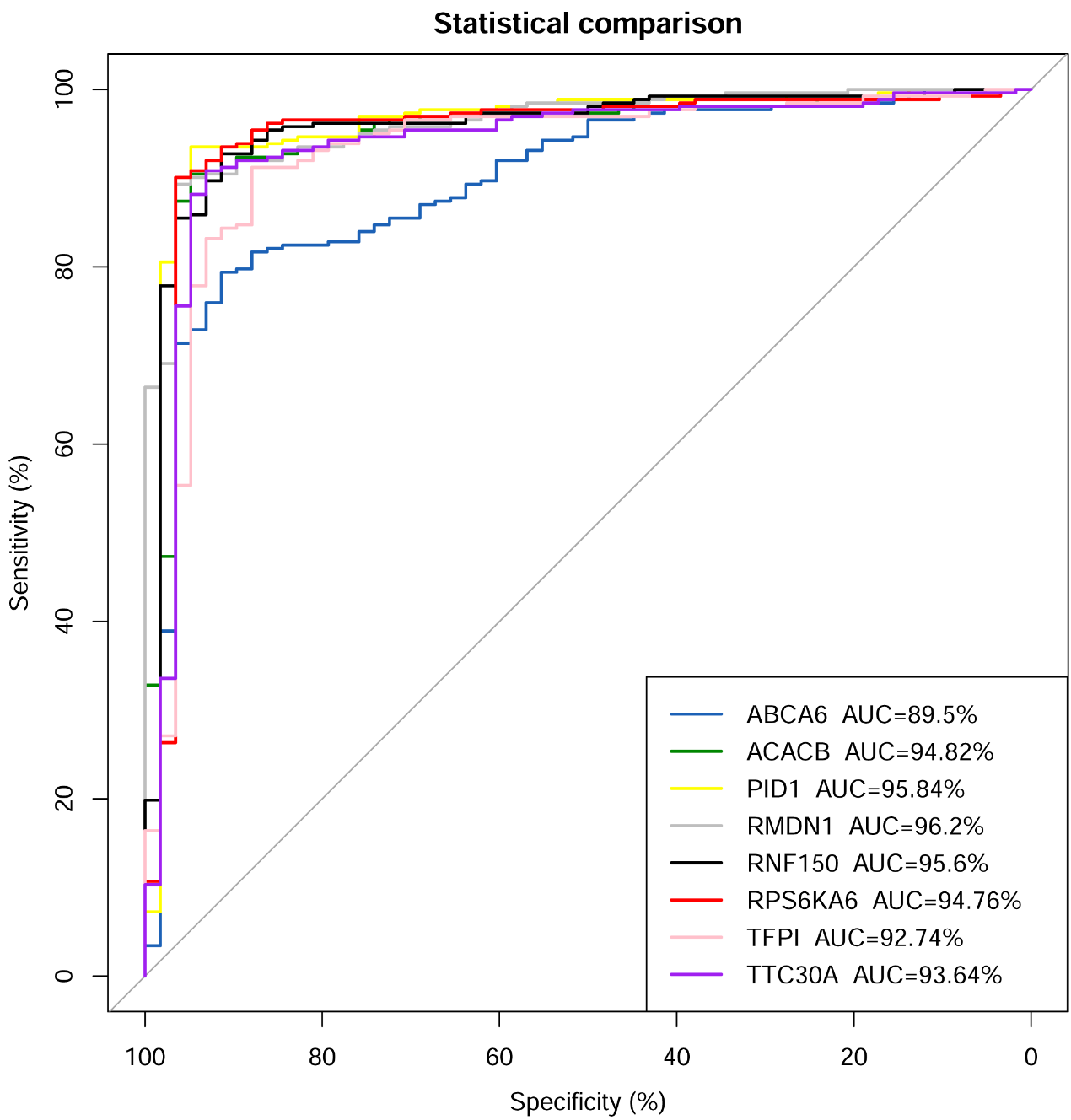


7B ROC curves of eight hub genes that received more attention


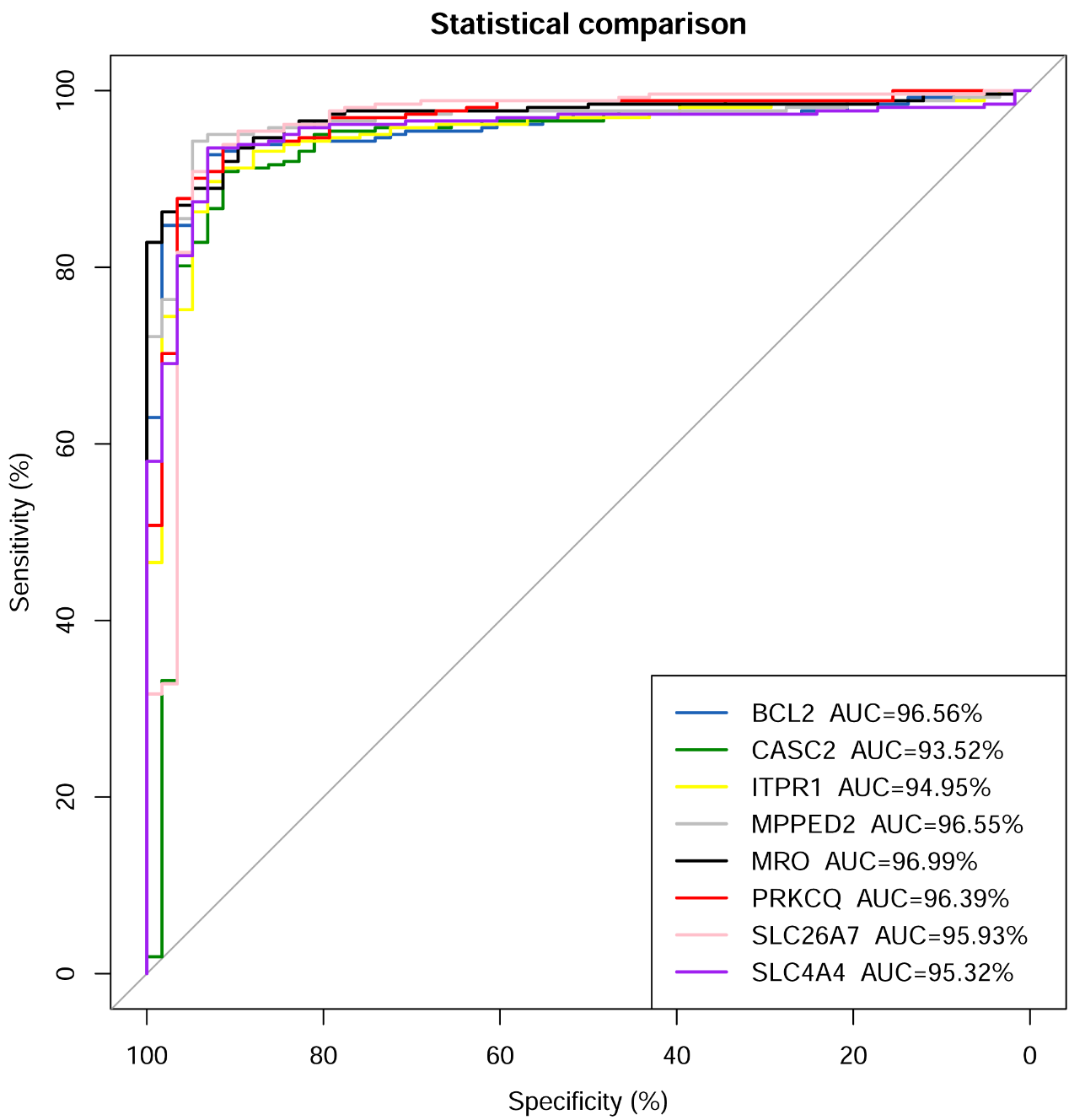


**Additional file Figure 8** Boxplot for methylation analysis of 8 hub genes.

**ABCA6**

**
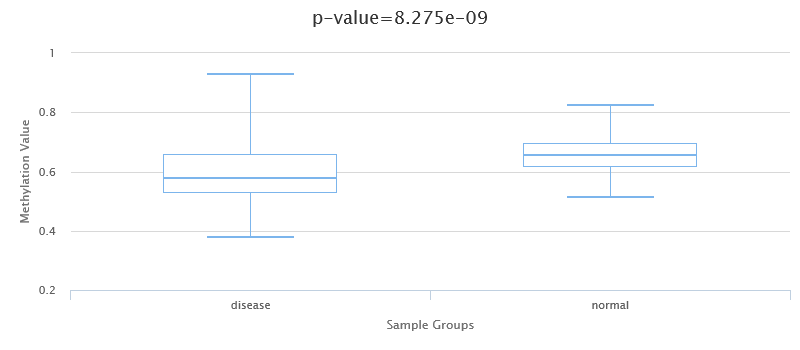
**

**ACACB**

**
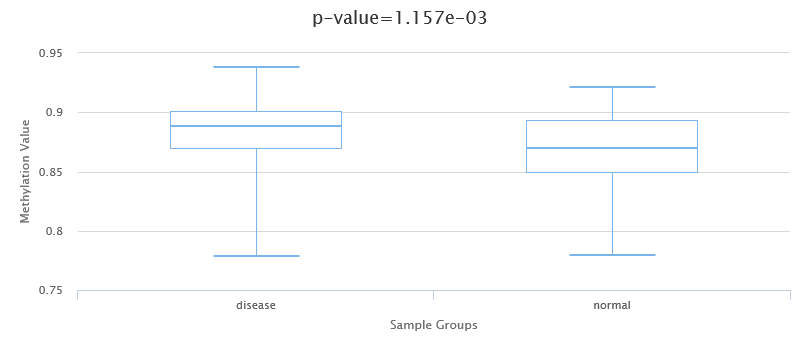
**

**PID1**

**
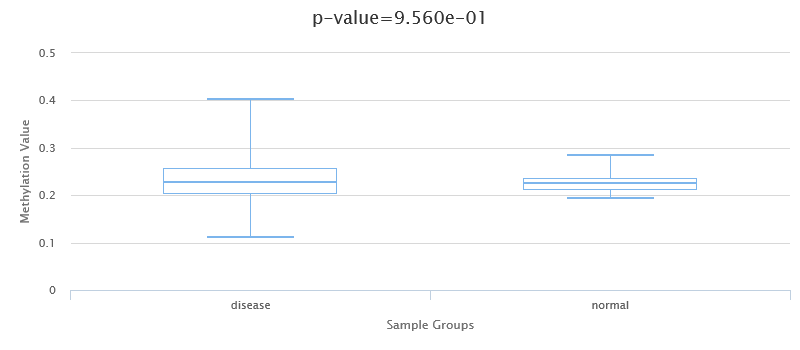
**

**RMDN1**

**
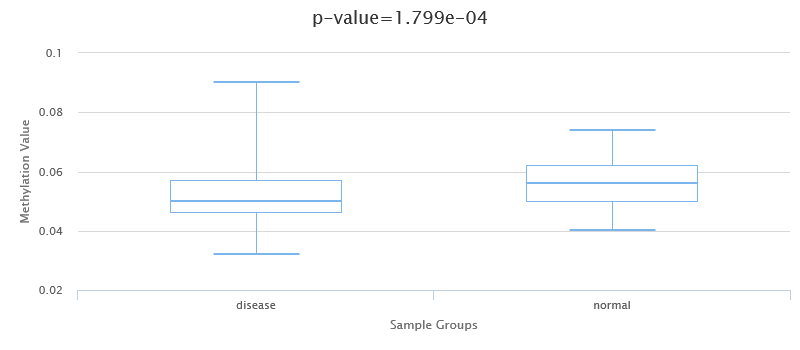
**

**RNF150**

**
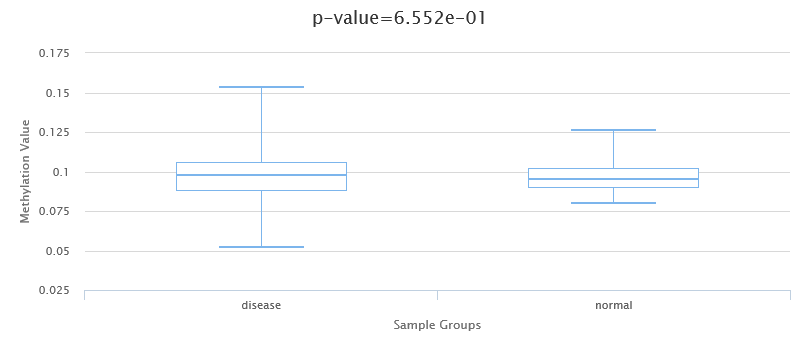
**

**RPS6KA6**

**
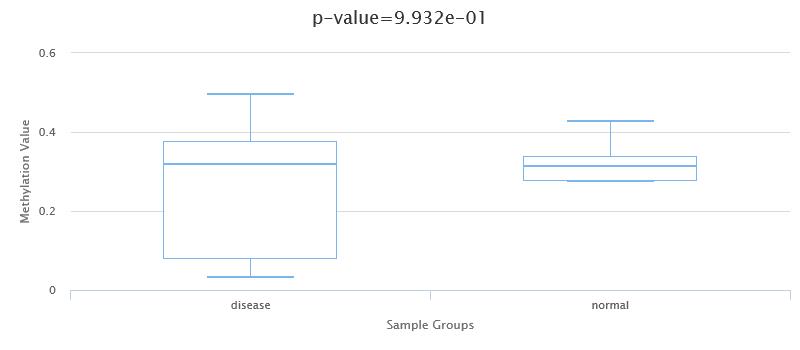
**

**TFPI**

**
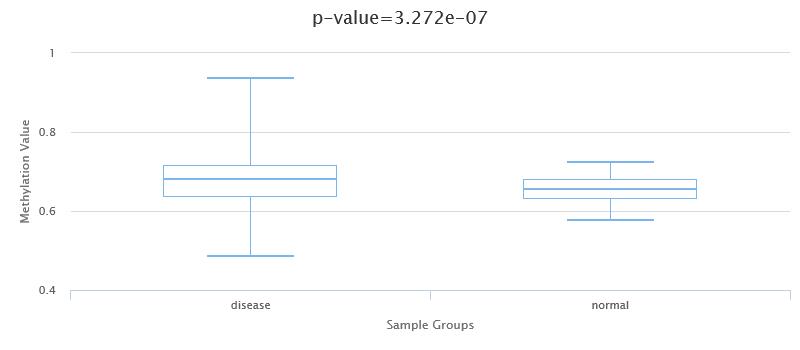
**

**TTC30A**

**
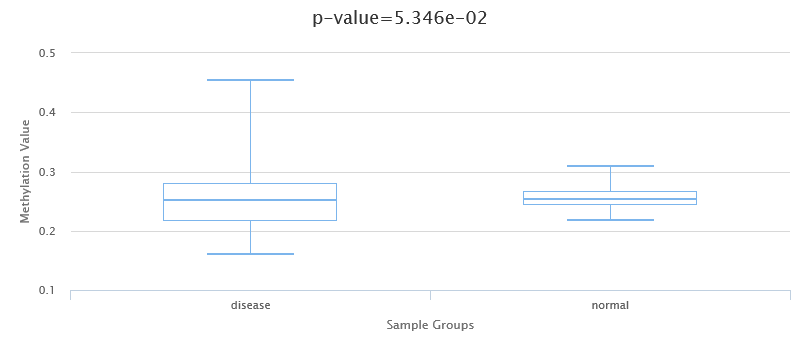
**

**Additional file Figure 9A-D** Association of Methylation sites with expression of 4 differentially methylated genes (DMGs).

Note:  The samples are sorted the genes expression levels and the samples without expression data were removed. On the right side, Pearson s correlation coefficients and P values from Wilcoxon rank-sum test for methylation sites and query gene expression are shown the expression of genes and DNA methylation are negatively correlated.

ABCA6


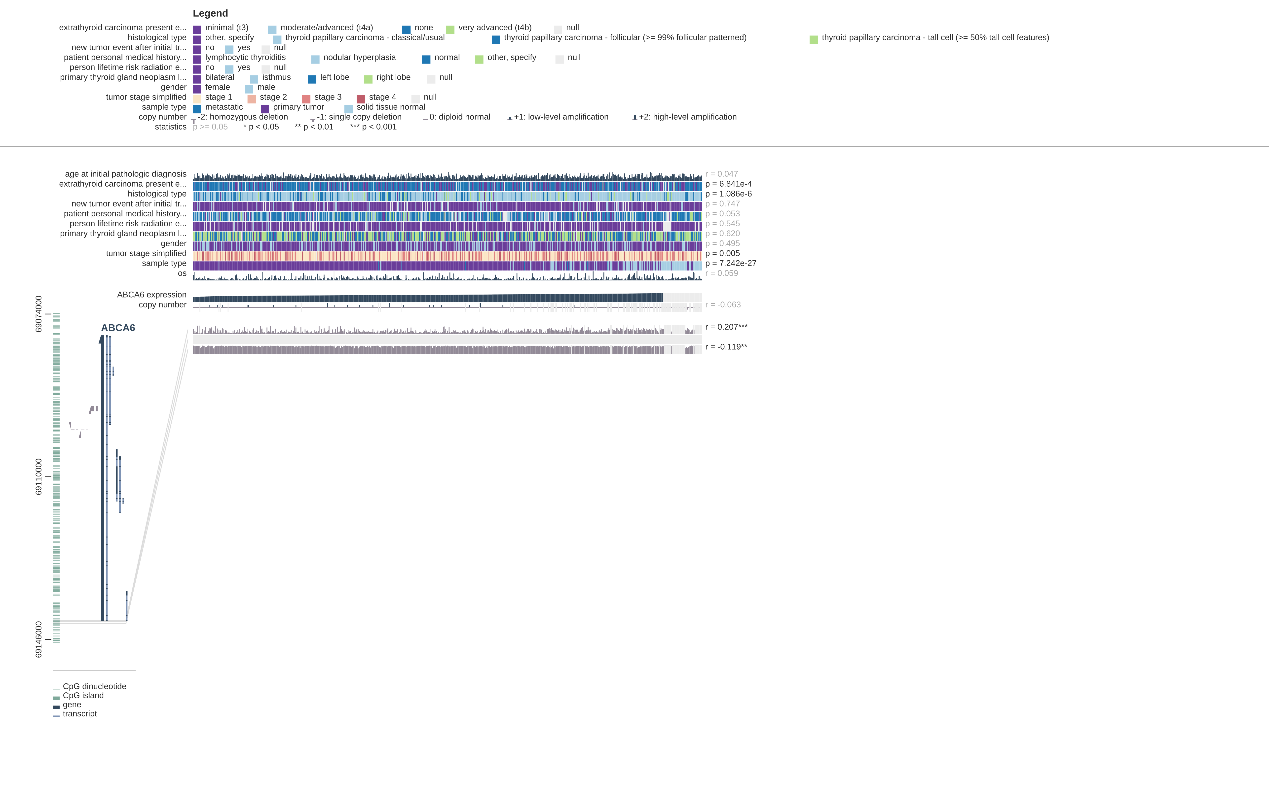


TFPI


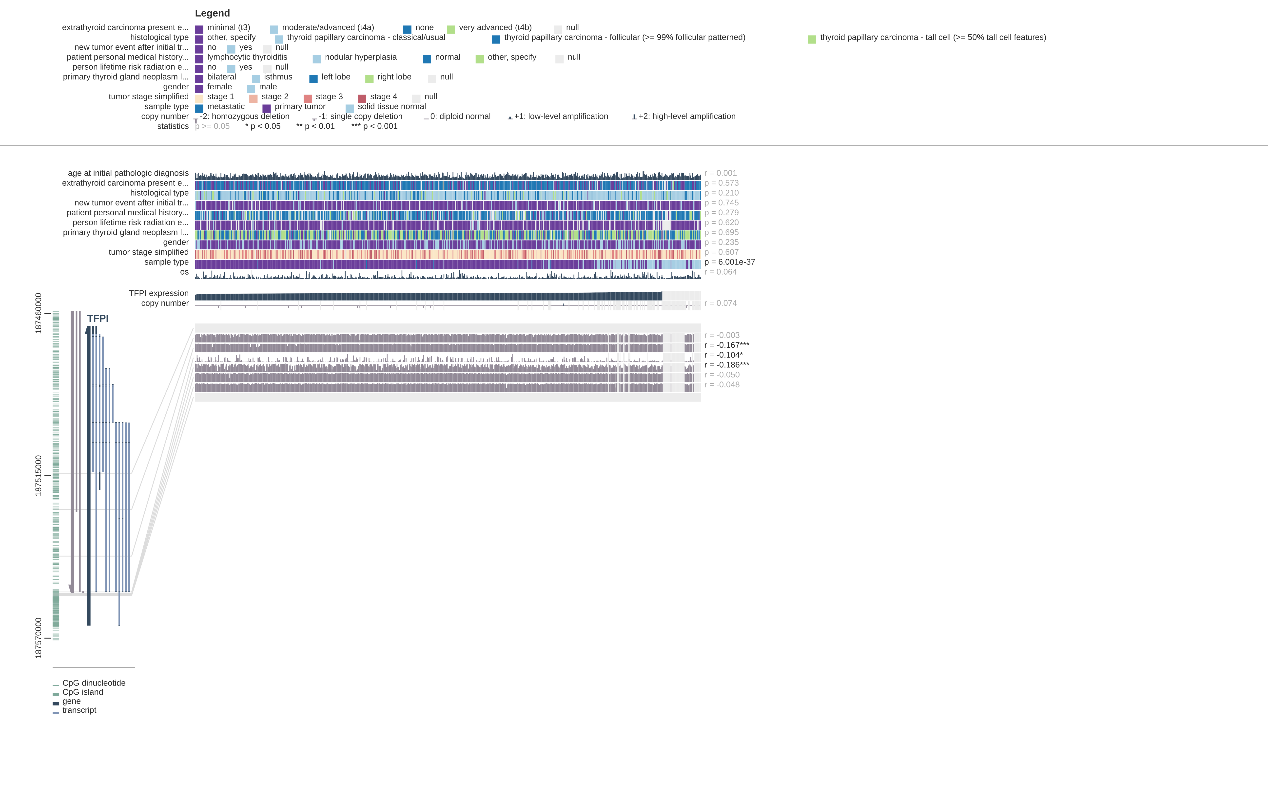


ACACB


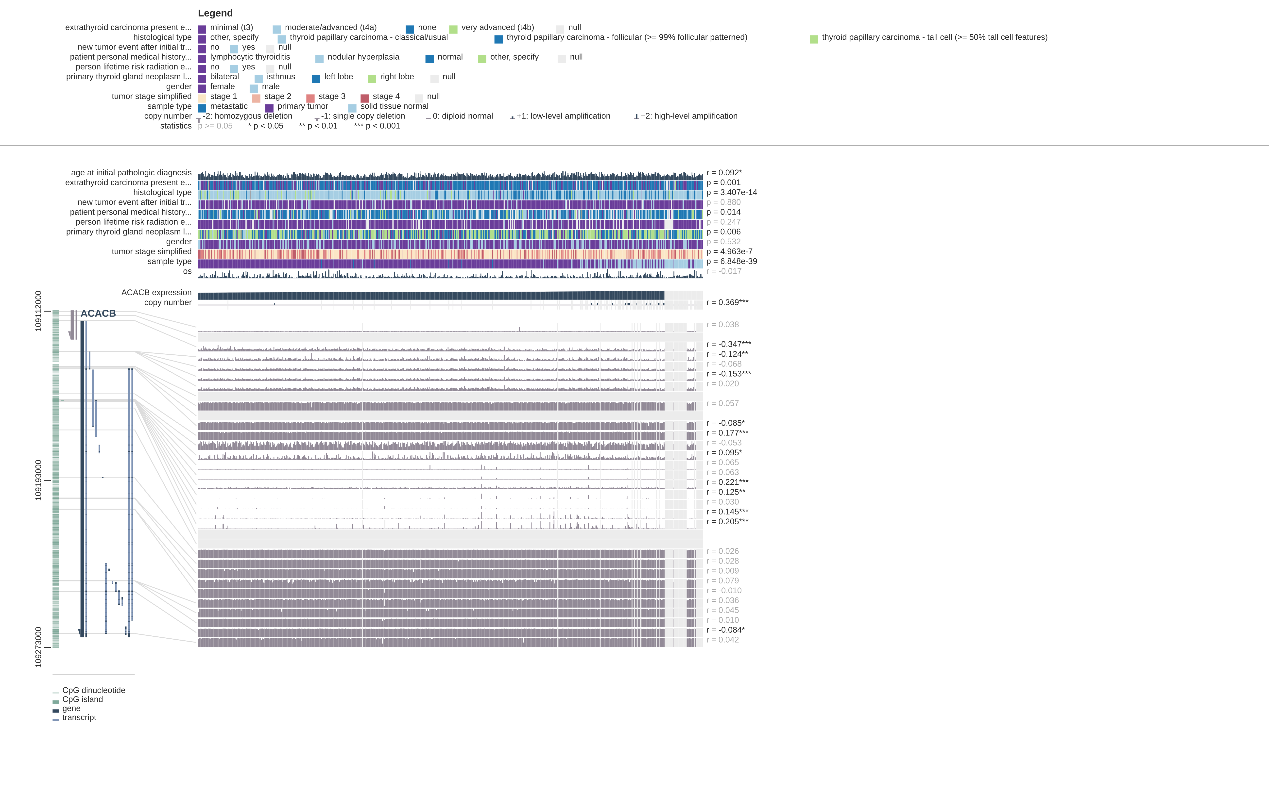


RMDN1

**
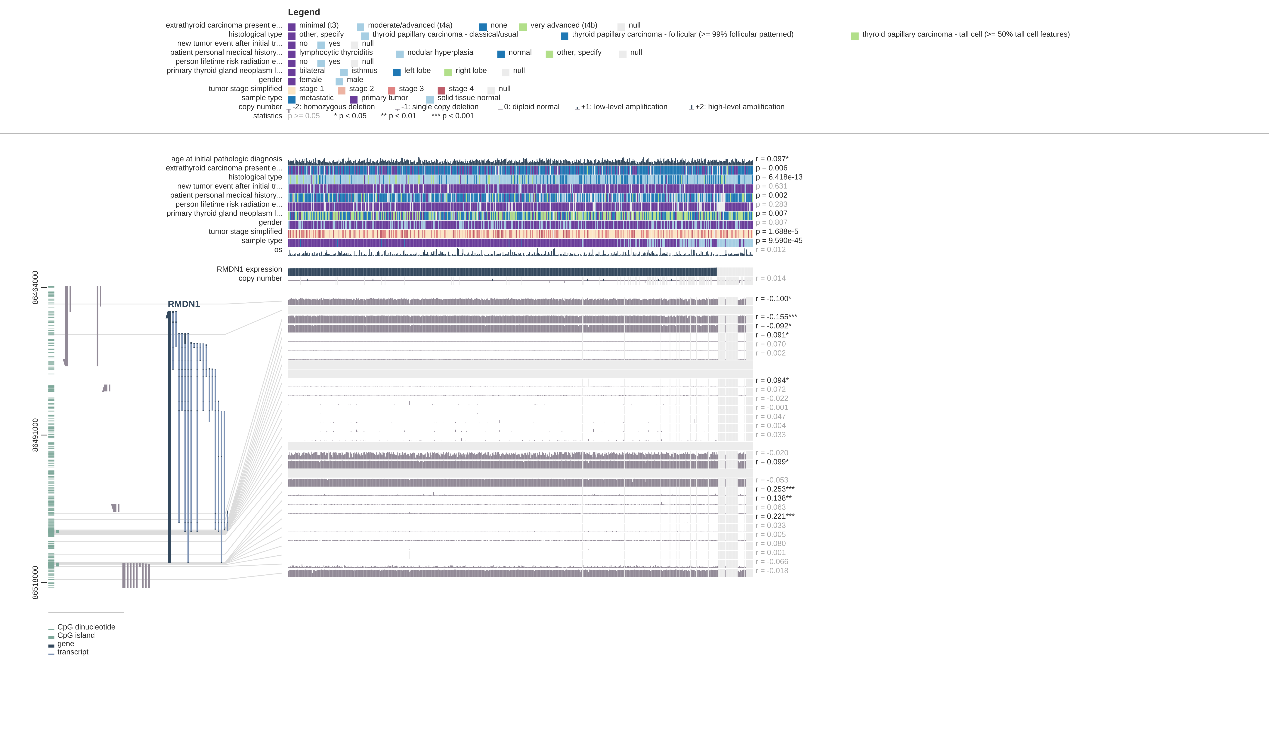
**

**Additional file Figure 10** The relationship between hub gene expression and tumor purity and tumor infiltrating immune cells.

**A**


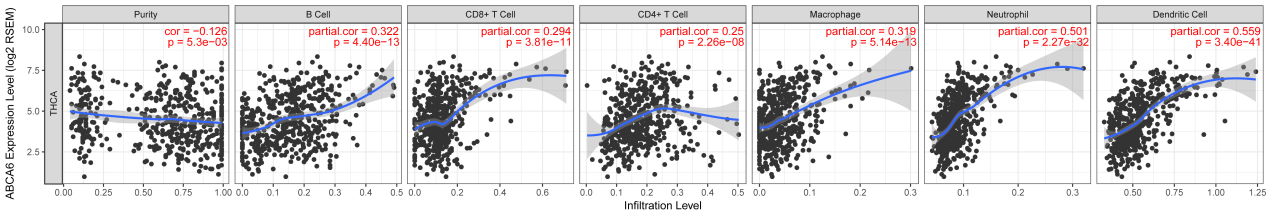


**B**


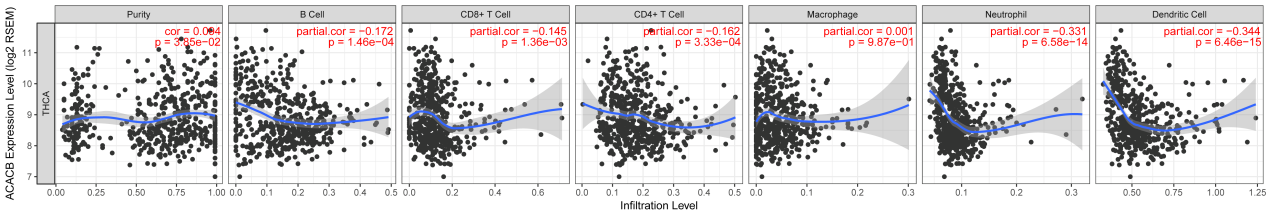


**C**

**
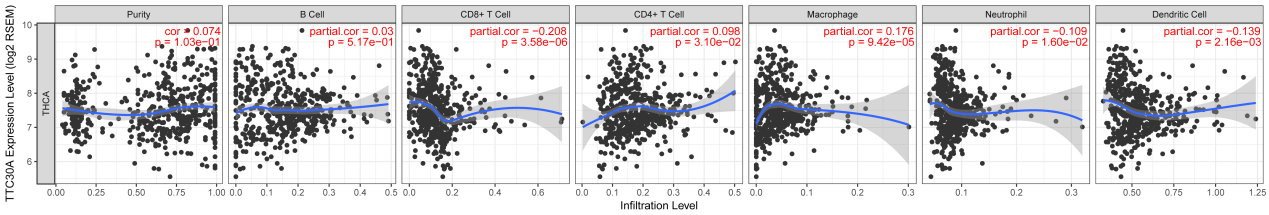
**

**D**

**
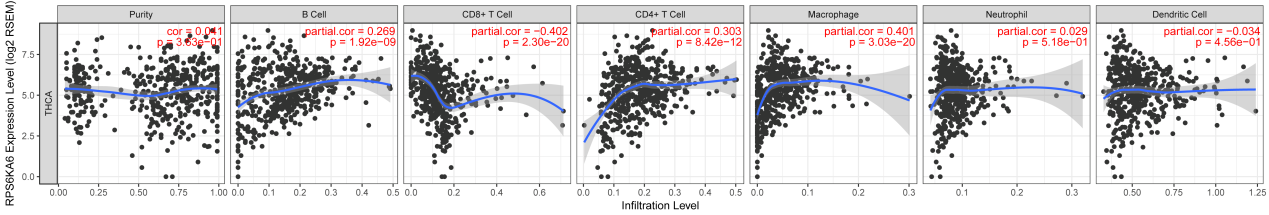
**

**E**

**
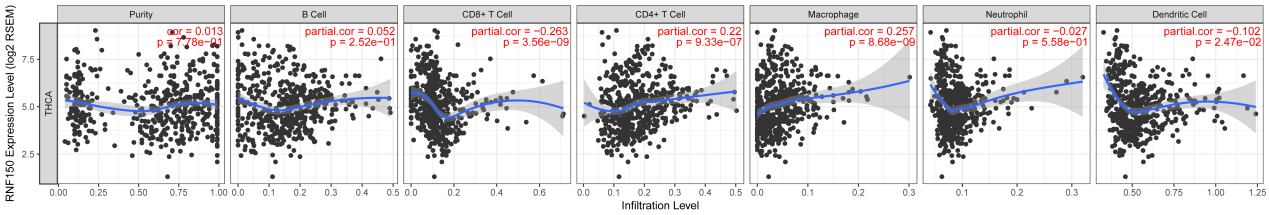
**

**F**

**
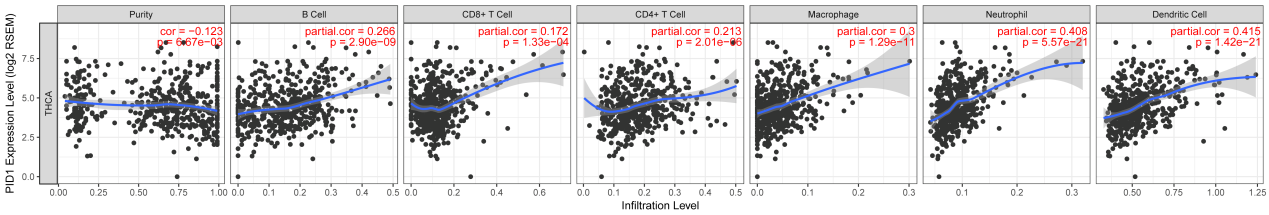
**

**G**

**
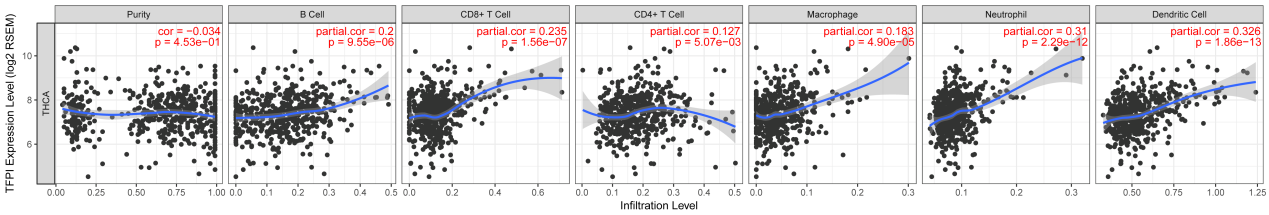
**

**Note:** Each black node represents a sample, where A indicates ABCA6, B indicates ACACB, C indicates TTC30A, D indicates RPS6KA6, E indicates RNF150, F indicates PID1, and G indicates TFPI.
