# Supplementary material for: Identification of hub genes in papillary thyroid carcinoma: robust rank aggregation and weighted gene co-expression network analysis
Source: J Transl Med. 2020 Apr 16;18:170. doi: 10.1186/s12967-020-02327-7 (PMC7161219; doi:10.1186/s12967-020-02327-7)
Supplement: Supplementary file 2 — Additional file 2: Table S1. Details of the six databases. [file 12967_2020_2327_MOESM2_ESM.docx]

**Additional file Table S1** Details of the six databases

| **Dataset** | **ID_REF** | **Type of thyroid tissue** | **histological subtype** | **Age** |
| --- | --- | --- | --- | --- |
| GSE6004 | GSM139002 | Normal Thyroid | NA | 71 |
| GSE6004 | GSM139003 | Normal Thyroid | NA | 34 |
| GSE6004 | GSM139004 | Normal Thyroid | NA | 29 |
| GSE6004 | GSM139005 | Normal Thyroid | NA | 21 |
| GSE6004 | GSM139006 | Papillary Thyroid Carcinoma | NA | 71 |
| GSE6004 | GSM139007 | Papillary Thyroid Carcinoma | NA | 34 |
| GSE6004 | GSM139008 | Papillary Thyroid Carcinoma | NA | 29 |
| GSE6004 | GSM139009 | Papillary Thyroid Carcinoma | NA | 21 |
| GSE6004 | GSM139010 | Papillary Thyroid Carcinoma | NA | 30 |
| GSE6004 | GSM139011 | Papillary Thyroid Carcinoma | NA | 45 |
| GSE6004 | GSM139012 | Papillary Thyroid Carcinoma | NA | 42 |
| GSE58545 | GSM1413225 | Normal thyroid | NA | 71 |
| GSE58545 | GSM1413226 | Normal thyroid | NA | 18 |
| GSE58545 | GSM1413227 | Papillary thyroid carcinoma | NA | 47 |
| GSE58545 | GSM1413228 | Normal thyroid | NA | 47 |
| GSE58545 | GSM1413229 | Papillary thyroid carcinoma | NA | 37 |
| GSE58545 | GSM1413230 | Normal thyroid | NA | 37 |
| GSE58545 | GSM1413231 | Papillary thyroid carcinoma | NA | 67 |
| GSE58545 | GSM1413232 | Normal thyroid | NA | 67 |
| GSE58545 | GSM1413233 | Normal thyroid | NA | 24 |
| GSE58545 | GSM1413234 | Normal thyroid | NA | 32 |
| GSE58545 | GSM1413235 | Normal thyroid | NA | 35 |
| GSE58545 | GSM1413236 | Papillary thyroid carcinoma | NA | 26 |
| GSE58545 | GSM1413237 | Papillary thyroid carcinoma | NA | 32 |
| GSE58545 | GSM1413238 | Papillary thyroid carcinoma | NA | 61 |
| GSE58545 | GSM1413239 | Normal thyroid | NA | 61 |
| GSE58545 | GSM1413240 | Normal thyroid | NA | 28 |
| GSE58545 | GSM1413241 | Papillary thyroid carcinoma | NA | 28 |
| GSE58545 | GSM1413242 | Normal thyroid | NA | 16 |
| GSE58545 | GSM1413243 | Normal thyroid | NA | 12 |
| GSE58545 | GSM1413244 | Papillary thyroid carcinoma | NA | 12 |
| GSE58545 | GSM1413245 | Normal thyroid | NA | 42 |
| GSE58545 | GSM1413246 | Papillary thyroid carcinoma | NA | 69 |
| GSE58545 | GSM1413247 | Normal thyroid | NA | 69 |
| GSE58545 | GSM1413248 | Normal thyroid | NA | 6 |
| GSE58545 | GSM1413249 | Papillary thyroid carcinoma | NA | 6 |
| GSE58545 | GSM1413250 | Normal thyroid | NA | 11 |
| GSE58545 | GSM1413251 | Papillary thyroid carcinoma | NA | 24 |
| GSE58545 | GSM1413252 | Normal thyroid | NA | 41 |
| GSE58545 | GSM1413253 | Papillary thyroid carcinoma | NA | 31 |
| GSE58545 | GSM1413254 | Normal thyroid | NA | 19 |
| GSE58545 | GSM1413255 | Papillary thyroid carcinoma | NA | 71 |
| GSE58545 | GSM1413256 | Papillary thyroid carcinoma | NA | 20 |
| GSE58545 | GSM1413257 | Papillary thyroid carcinoma | NA | 66 |
| GSE58545 | GSM1413258 | Papillary thyroid carcinoma | NA | 21 |
| GSE58545 | GSM1413259 | Papillary thyroid carcinoma | NA | 71 |
| GSE58545 | GSM1413260 | Papillary thyroid carcinoma | NA | 44 |
| GSE58545 | GSM1413261 | Papillary thyroid carcinoma | NA | 31 |
| GSE58545 | GSM1413262 | Papillary thyroid carcinoma | NA | 28 |
| GSE58545 | GSM1413263 | Papillary thyroid carcinoma | NA | 44 |
| GSE58545 | GSM1413264 | Papillary thyroid carcinoma | NA | 30 |
| GSE58545 | GSM1413265 | Papillary thyroid carcinoma | NA | 21 |
| GSE58545 | GSM1413266 | Papillary thyroid carcinoma | NA | 19 |
| GSE58545 | GSM1413267 | Papillary thyroid carcinoma | NA | 59 |
| GSE58545 | GSM1413268 | Papillary thyroid carcinoma | NA | 64 |
| GSE58545 | GSM1413269 | Papillary thyroid carcinoma | NA | 23 |
| GSE27155 | GSM671268 | Normal Thyroid | NA | NA |
| GSE27155 | GSM671269 | Normal Thyroid | NA | NA |
| GSE27155 | GSM671270 | Normal Thyroid | NA | NA |
| GSE27155 | GSM671271 | Normal Thyroid | NA | NA |
| GSE27155 | GSM671297 | Papillary Thyroid Carcinoma | follicular | NA |
| GSE27155 | GSM671298 | Papillary Thyroid Carcinoma | follicular | NA |
| GSE27155 | GSM671299 | Papillary Thyroid Carcinoma | follicular | NA |
| GSE27155 | GSM671300 | Papillary Thyroid Carcinoma | follicular | NA |
| GSE27155 | GSM671301 | Papillary Thyroid Carcinoma | follicular | NA |
| GSE27155 | GSM671302 | Papillary Thyroid Carcinoma | tall cell | NA |
| GSE27155 | GSM671303 | Papillary Thyroid Carcinoma | follicular | NA |
| GSE27155 | GSM671304 | Papillary Thyroid Carcinoma | classical type | NA |
| GSE27155 | GSM671305 | Papillary Thyroid Carcinoma | classical type | NA |
| GSE27155 | GSM671306 | Papillary Thyroid Carcinoma | classical type | NA |
| GSE27155 | GSM671307 | Papillary Thyroid Carcinoma | classical type | NA |
| GSE27155 | GSM671308 | Papillary Thyroid Carcinoma | classical type | NA |
| GSE27155 | GSM671309 | Papillary Thyroid Carcinoma | classical type | NA |
| GSE27155 | GSM671310 | Papillary Thyroid Carcinoma | follicular | NA |
| GSE27155 | GSM671311 | Papillary Thyroid Carcinoma | classical type | NA |
| GSE27155 | GSM671312 | Papillary Thyroid Carcinoma | classical type | NA |
| GSE27155 | GSM671313 | Papillary Thyroid Carcinoma | classical type | NA |
| GSE27155 | GSM671314 | Papillary Thyroid Carcinoma | classical type | NA |
| GSE27155 | GSM671315 | Papillary Thyroid Carcinoma | classical type | NA |
| GSE27155 | GSM671316 | Papillary Thyroid Carcinoma | classical type | NA |
| GSE27155 | GSM671317 | Papillary Thyroid Carcinoma | classical type | NA |
| GSE27155 | GSM671318 | Papillary Thyroid Carcinoma | classical type | NA |
| GSE27155 | GSM671319 | Papillary Thyroid Carcinoma | classical type | NA |
| GSE27155 | GSM671320 | Papillary Thyroid Carcinoma | tall cell | NA |
| GSE27155 | GSM671321 | Papillary Thyroid Carcinoma | classical type | NA |
| GSE27155 | GSM671322 | Papillary Thyroid Carcinoma | tall cell | NA |
| GSE27155 | GSM671323 | Papillary Thyroid Carcinoma | classical type | NA |
| GSE27155 | GSM671324 | Papillary Thyroid Carcinoma | tall cell | NA |
| GSE27155 | GSM671325 | Papillary Thyroid Carcinoma | tall cell | NA |
| GSE27155 | GSM671326 | Papillary Thyroid Carcinoma | follicular | NA |
| GSE27155 | GSM671327 | Papillary Thyroid Carcinoma | tall cell | NA |
| GSE27155 | GSM671328 | Papillary Thyroid Carcinoma | classical type | NA |
| GSE27155 | GSM671329 | Papillary Thyroid Carcinoma | classical type | NA |
| GSE27155 | GSM671330 | Papillary Thyroid Carcinoma | classical type | NA |
| GSE27155 | GSM671331 | Papillary Thyroid Carcinoma | classical type | NA |
| GSE27155 | GSM671332 | Papillary Thyroid Carcinoma | tall cell | NA |
| GSE27155 | GSM671333 | Papillary Thyroid Carcinoma | tall cell | NA |
| GSE27155 | GSM671334 | Papillary Thyroid Carcinoma | classical type | NA |
| GSE27155 | GSM671335 | Papillary Thyroid Carcinoma | classical type | NA |
| GSE27155 | GSM671336 | Papillary Thyroid Carcinoma | tall cell | NA |
| GSE27155 | GSM671337 | Papillary Thyroid Carcinoma | classical type | NA |
| GSE27155 | GSM671338 | Papillary Thyroid Carcinoma | classical type | NA |
| GSE27155 | GSM671339 | Papillary Thyroid Carcinoma | tall cell | NA |
| GSE27155 | GSM671340 | Papillary Thyroid Carcinoma | follicular | NA |
| GSE27155 | GSM671341 | Papillary Thyroid Carcinoma | follicular | NA |
| GSE27155 | GSM671342 | Papillary Thyroid Carcinoma | follicular | NA |
| GSE27155 | GSM671343 | Papillary Thyroid Carcinoma | follicular | NA |
| GSE27155 | GSM671344 | Papillary Thyroid Carcinoma | follicular | NA |
| GSE27155 | GSM671345 | Papillary Thyroid Carcinoma | follicular | NA |
| GSE27155 | GSM671346 | Papillary Thyroid Carcinoma | follicular | NA |
| GSE27155 | GSM671347 | Papillary Thyroid Carcinoma | classical type | NA |
| GSE53157 | GSM1283132 | Papillary Thyroid Carcinoma | NA | 26 |
| GSE53157 | GSM1283133 | Papillary Thyroid Carcinoma | NA | 31 |
| GSE53157 | GSM1283134 | Papillary Thyroid Carcinoma | NA | 38 |
| GSE53157 | GSM1283135 | Papillary Thyroid Carcinoma | NA | 35 |
| GSE53157 | GSM1283136 | Papillary Thyroid Carcinoma | NA | 42 |
| GSE53157 | GSM1283137 | Papillary Thyroid Carcinoma | NA | 64 |
| GSE53157 | GSM1283138 | Papillary Thyroid Carcinoma | NA | 14 |
| GSE53157 | GSM1283139 | Normal Thyroid | NA | 46 |
| GSE53157 | GSM1283140 | Normal Thyroid | NA | 38 |
| GSE60542 | GSM1481838 | Normal Thyroid | Trabecular | 52 |
| GSE60542 | GSM1481839 | Papillary Thyroid Carcinoma | Papillary | 35 |
| GSE60542 | GSM1481841 | Papillary Thyroid Carcinoma | Trabecular | 29 |
| GSE60542 | GSM1481843 | Normal Thyroid | Papillary | 37 |
| GSE60542 | GSM1481845 | Normal Thyroid | Papillary | 26 |
| GSE60542 | GSM1481846 | Papillary Thyroid Carcinoma | Papillary | 26 |
| GSE60542 | GSM1481848 | Normal Thyroid | Papillary | 57 |
| GSE60542 | GSM1481849 | Papillary Thyroid Carcinoma | Papillary | 57 |
| GSE60542 | GSM1481851 | Normal Thyroid | Papillary | 41 |
| GSE60542 | GSM1481852 | Normal Thyroid | Follicular | 39 |
| GSE60542 | GSM1481854 | Papillary Thyroid Carcinoma | Papillary | 37 |
| GSE60542 | GSM1481858 | Papillary Thyroid Carcinoma | Follicular | 39 |
| GSE60542 | GSM1481861 | Papillary Thyroid Carcinoma | Papillary | 25 |
| GSE60542 | GSM1481862 | Normal Thyroid | Follicular | 51 |
| GSE60542 | GSM1481863 | Papillary Thyroid Carcinoma | Follicular | 51 |
| GSE60542 | GSM1481865 | Papillary Thyroid Carcinoma | Papillary | 42 |
| GSE60542 | GSM1481867 | Normal Thyroid | Follicular | 79 |
| GSE60542 | GSM1481868 | Papillary Thyroid Carcinoma | Follicular | 79 |
| GSE60542 | GSM1481872 | Papillary Thyroid Carcinoma | Papillary | 73 |
| GSE60542 | GSM1481875 | Papillary Thyroid Carcinoma | Follicular | 39 |
| GSE60542 | GSM1481876 | Normal Thyroid | Papillary | 14 |
| GSE60542 | GSM1481877 | Normal Thyroid | Papillary | 50 |
| GSE60542 | GSM1481880 | Normal Thyroid | Papillary | 42 |
| GSE60542 | GSM1481882 | Papillary Thyroid Carcinoma | Tubulair | 38 |
| GSE60542 | GSM1481883 | Papillary Thyroid Carcinoma | poorly differentiated with well differentiated PTC compatible areas | 71 |
| GSE60542 | GSM1481888 | Papillary Thyroid Carcinoma | Papillary | 50 |
| GSE60542 | GSM1481889 | Papillary Thyroid Carcinoma | Papillary | 14 |
| GSE60542 | GSM1481890 | Papillary Thyroid Carcinoma | Papillary | 59 |
| GSE60542 | GSM1481891 | Normal Thyroid | Papillary | 46 |
| GSE60542 | GSM1481893 | Normal Thyroid | Papillary | 25 |
| GSE60542 | GSM1481894 | Normal Thyroid | Papillary | 73 |
| GSE60542 | GSM1481895 | Normal Thyroid | Follicular | 44 |
| GSE60542 | GSM1481896 | Normal Thyroid | Papillary | 33 |
| GSE60542 | GSM1481898 | Papillary Thyroid Carcinoma | Papillary | 46 |
| GSE60542 | GSM1481900 | Normal Thyroid | Follicular | 39 |
| GSE60542 | GSM1481901 | Normal Thyroid | Papillary | 50 |
| GSE60542 | GSM1481902 | Papillary Thyroid Carcinoma | Follicular | 44 |
| GSE60542 | GSM1481903 | Papillary Thyroid Carcinoma | Papillary | 33 |
| GSE60542 | GSM1481904 | Normal Thyroid | Papillary | 12.5 |
| GSE60542 | GSM1481906 | Papillary Thyroid Carcinoma | Papillary | 50 |
| GSE60542 | GSM1481911 | Normal Thyroid | Papillary | 43 |
| GSE60542 | GSM1481912 | Papillary Thyroid Carcinoma | Trabecular | 52 |
| GSE60542 | GSM1481914 | Papillary Thyroid Carcinoma | Papillary | 43 |
| GSE60542 | GSM1481915 | Papillary Thyroid Carcinoma | Papillary | 65 |
| GSE60542 | GSM1481916 | Normal Thyroid | Trabecular | 29 |
| GSE60542 | GSM1481918 | Normal Thyroid | Papillary | 65 |
| GSE60542 | GSM1481919 | Normal Thyroid | Papillary | 47 |
| GSE60542 | GSM1481920 | Normal Thyroid | Papillary | 59 |
| GSE60542 | GSM1481921 | Normal Thyroid | Diffuse sclerosis | 36 |
| GSE60542 | GSM1481922 | Normal Thyroid | Follicular | 34 |
| GSE60542 | GSM1481923 | Papillary Thyroid Carcinoma | Papillary | 47 |
| GSE60542 | GSM1481924 | Papillary Thyroid Carcinoma | Papillary | 59 |
| GSE60542 | GSM1481926 | Papillary Thyroid Carcinoma | Diffuse sclerosis | 36 |
| GSE60542 | GSM1481929 | Papillary Thyroid Carcinoma | Follicular | 34 |
| GSE33630 | GSM831759 | Papillary thyroid carcinoma | NA | NA |
| GSE33630 | GSM831760 | Papillary thyroid carcinoma | NA | NA |
| GSE33630 | GSM831761 | Papillary thyroid carcinoma | NA | NA |
| GSE33630 | GSM831762 | Papillary thyroid carcinoma | NA | NA |
| GSE33630 | GSM831763 | Papillary thyroid carcinoma | NA | NA |
| GSE33630 | GSM831764 | Papillary thyroid carcinoma | NA | NA |
| GSE33630 | GSM831765 | Papillary thyroid carcinoma | NA | NA |
| GSE33630 | GSM831766 | Papillary thyroid carcinoma | NA | NA |
| GSE33630 | GSM831767 | Papillary thyroid carcinoma | NA | NA |
| GSE33630 | GSM831768 | Papillary thyroid carcinoma | NA | NA |
| GSE33630 | GSM831769 | Papillary thyroid carcinoma | NA | NA |
| GSE33630 | GSM831770 | Papillary thyroid carcinoma | NA | NA |
| GSE33630 | GSM831771 | Papillary thyroid carcinoma | NA | NA |
| GSE33630 | GSM831772 | Papillary thyroid carcinoma | NA | NA |
| GSE33630 | GSM831773 | Papillary thyroid carcinoma | NA | NA |
| GSE33630 | GSM831774 | Papillary thyroid carcinoma | NA | NA |
| GSE33630 | GSM831775 | Papillary thyroid carcinoma | NA | NA |
| GSE33630 | GSM831776 | Papillary thyroid carcinoma | NA | NA |
| GSE33630 | GSM831777 | Papillary thyroid carcinoma | NA | NA |
| GSE33630 | GSM831778 | Papillary thyroid carcinoma | NA | NA |
| GSE33630 | GSM831779 | Papillary thyroid carcinoma | NA | NA |
| GSE33630 | GSM831780 | Papillary thyroid carcinoma | NA | NA |
| GSE33630 | GSM831781 | Papillary thyroid carcinoma | NA | NA |
| GSE33630 | GSM831782 | Papillary thyroid carcinoma | NA | NA |
| GSE33630 | GSM831783 | Papillary thyroid carcinoma | NA | NA |
| GSE33630 | GSM831784 | Papillary thyroid carcinoma | NA | NA |
| GSE33630 | GSM831785 | Papillary thyroid carcinoma | NA | NA |
| GSE33630 | GSM831786 | Papillary thyroid carcinoma | NA | NA |
| GSE33630 | GSM831787 | Papillary thyroid carcinoma | NA | NA |
| GSE33630 | GSM831788 | Papillary thyroid carcinoma | NA | NA |
| GSE33630 | GSM831789 | Papillary thyroid carcinoma | NA | NA |
| GSE33630 | GSM831790 | Papillary thyroid carcinoma | NA | NA |
| GSE33630 | GSM831791 | Papillary thyroid carcinoma | NA | NA |
| GSE33630 | GSM831792 | Papillary thyroid carcinoma | NA | NA |
| GSE33630 | GSM831793 | Papillary thyroid carcinoma | NA | NA |
| GSE33630 | GSM831794 | Papillary thyroid carcinoma | NA | NA |
| GSE33630 | GSM831795 | Papillary thyroid carcinoma | NA | NA |
| GSE33630 | GSM831796 | Papillary thyroid carcinoma | NA | NA |
| GSE33630 | GSM831797 | Papillary thyroid carcinoma | NA | NA |
| GSE33630 | GSM831798 | Papillary thyroid carcinoma | NA | NA |
| GSE33630 | GSM831799 | Papillary thyroid carcinoma | NA | NA |
| GSE33630 | GSM831800 | Papillary thyroid carcinoma | NA | NA |
| GSE33630 | GSM831801 | Papillary thyroid carcinoma | NA | NA |
| GSE33630 | GSM831802 | Papillary thyroid carcinoma | NA | NA |
| GSE33630 | GSM831803 | Papillary thyroid carcinoma | NA | NA |
| GSE33630 | GSM831804 | Papillary thyroid carcinoma | NA | NA |
| GSE33630 | GSM831805 | Papillary thyroid carcinoma | NA | NA |
| GSE33630 | GSM831806 | Papillary thyroid carcinoma | NA | NA |
| GSE33630 | GSM831807 | Papillary thyroid carcinoma | NA | NA |
| GSE33630 | GSM831808 | Normal Thyroid | NA | NA |
| GSE33630 | GSM831809 | Normal Thyroid | NA | NA |
| GSE33630 | GSM831810 | Normal Thyroid | NA | NA |
| GSE33630 | GSM831811 | Normal Thyroid | NA | NA |
| GSE33630 | GSM831812 | Normal Thyroid | NA | NA |
| GSE33630 | GSM831813 | Normal Thyroid | NA | NA |
| GSE33630 | GSM831814 | Normal Thyroid | NA | NA |
| GSE33630 | GSM831815 | Normal Thyroid | NA | NA |
| GSE33630 | GSM831816 | Normal Thyroid | NA | NA |
| GSE33630 | GSM831817 | Normal Thyroid | NA | NA |
| GSE33630 | GSM831818 | Normal Thyroid | NA | NA |
| GSE33630 | GSM831819 | Normal Thyroid | NA | NA |
| GSE33630 | GSM831820 | Normal Thyroid | NA | NA |
| GSE33630 | GSM831821 | Normal Thyroid | NA | NA |
| GSE33630 | GSM831822 | Normal Thyroid | NA | NA |
| GSE33630 | GSM831823 | Normal Thyroid | NA | NA |
| GSE33630 | GSM831824 | Normal Thyroid | NA | NA |
| GSE33630 | GSM831825 | Normal Thyroid | NA | NA |
| GSE33630 | GSM831826 | Normal Thyroid | NA | NA |
| GSE33630 | GSM831827 | Normal Thyroid | NA | NA |
| GSE33630 | GSM831828 | Normal Thyroid | NA | NA |
| GSE33630 | GSM831829 | Normal Thyroid | NA | NA |
| GSE33630 | GSM831830 | Normal Thyroid | NA | NA |
| GSE33630 | GSM831831 | Normal Thyroid | NA | NA |
| GSE33630 | GSM831832 | Normal Thyroid | NA | NA |
| GSE33630 | GSM831833 | Normal Thyroid | NA | NA |
| GSE33630 | GSM831834 | Normal Thyroid | NA | NA |
| GSE33630 | GSM831835 | Normal Thyroid | NA | NA |
| GSE33630 | GSM831836 | Normal Thyroid | NA | NA |
| GSE33630 | GSM831837 | Normal Thyroid | NA | NA |
| GSE33630 | GSM831838 | Normal Thyroid | NA | NA |
| GSE33630 | GSM831839 | Normal Thyroid | NA | NA |
| GSE33630 | GSM831840 | Normal Thyroid | NA | NA |
| GSE33630 | GSM831841 | Normal Thyroid | NA | NA |
| GSE33630 | GSM831842 | Normal Thyroid | NA | NA |
| GSE33630 | GSM831843 | Normal Thyroid | NA | NA |
| GSE33630 | GSM831844 | Normal Thyroid | NA | NA |
| GSE33630 | GSM831845 | Normal Thyroid | NA | NA |
| GSE33630 | GSM831846 | Normal Thyroid | NA | NA |
| GSE33630 | GSM831847 | Normal Thyroid | NA | NA |
| GSE33630 | GSM831848 | Normal Thyroid | NA | NA |
| GSE33630 | GSM831849 | Normal Thyroid | NA | NA |
| GSE33630 | GSM831850 | Normal Thyroid | NA | NA |
| GSE33630 | GSM831851 | Normal Thyroid | NA | NA |
| GSE33630 | GSM831852 | Normal Thyroid | NA | NA |

**Note:** NA means missing data. The table information section is missing because the information provided by the datasets is incomplete.
